# Colloidal Plasmonic TiN Nanoparticles for Efficient Solar Seawater Desalination

**DOI:** 10.1021/acsami.3c13479

**Published:** 2023-11-20

**Authors:** Xiaopeng Bai, Shiu Hei Lam, Jingtian Hu, Ka Kit Chui, Xiao-Ming Zhu, Lei Shao, Tsz Him Chow, Jianfang Wang

**Affiliations:** † Department of Physics, The Chinese University of Hong Kong, Shatin, Hong Kong SAR 999077, China; ‡ State Key Laboratory of Quality Research in Chinese Medicine, Macau Institute for Applied Research in Medicine and Health, Macau University of Science and Technology, Avenida Wai Long, Taipa, Macao SAR 999078, China; § State Key Laboratory of Optoelectronic Materials and Technologies, Guangdong Province Key Laboratory of Display Material and Technology, School of Electronics and Information Technology, Sun Yat-sen University, Guangzhou 510275, China

**Keywords:** plasmon resonance, plasmonic nanoparticles, solar seawater desalination, TiN nanoparticles, TiN nanorod arrays

## Abstract

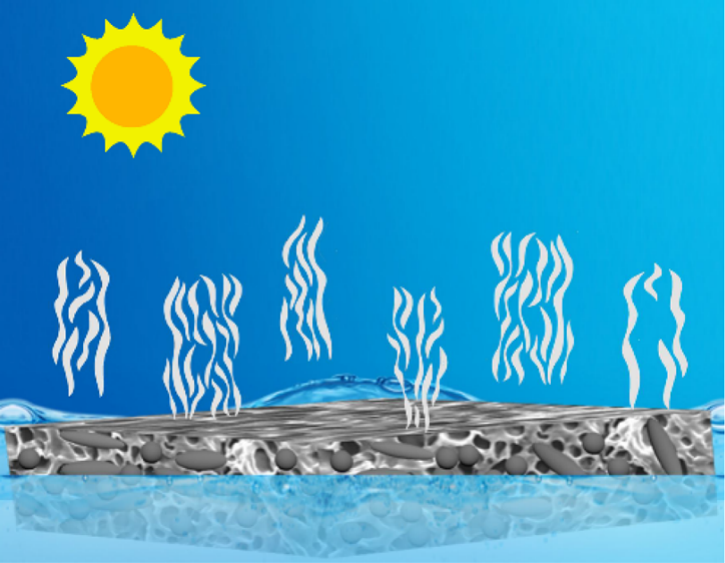

Transferring traditional plasmonic noble metal nanomaterials from the laboratory to industrial production has remained challenging due to the high price of noble metals. The development of cost-effective non-noble-metal alternatives with outstanding plasmonic properties has therefore become essential. Herein, we report on the gram-scale production of differently shaped TiN nanoparticles with strong plasmon-enabled broadband light absorption, including differently sized TiN nanospheres, nanobipyramids, and nanorod arrays. The TiN nanospheres and nanobipyramids are further coembedded in highly porous poly(vinyl alcohol) films to function as a photothermal material for solar seawater desalination. A seawater evaporation rate of 3.8 kg m^–2^ h^–1^ is achieved, which marks the record performance among all plasmonic solar seawater desalination systems reported so far. The removal percentage of phenol reaches 98.3%, which is attributed to the joint action of the excellent photocatalytic ability and the superhydrophilicity of the porous TiN-based composite film.

## 1 Introduction

Localized surface plasmon resonance (LSPR) refers to the electromagnetic resonance associated with the oscillations of free charge carriers within nanoparticles. LSPR endows nanoparticles with many attractive properties, such as highly controllable resonance wavelengths,^1^ large extinction cross sections,^2^ enhanced local electromagnetic field,^3^ and hot charge carrier generation.^4^ Numerous photodriven applications are based on LSPR, including solar water steaming,^5^ photocatalysis,^6^ photothermal therapy,^7^ and plasmonic color generation.^8^ Conventional plasmonic nanomaterials are mainly based on noble metals such as Au and Ag. They have been widely investigated.^1,9^ Their plasmon wavelengths can be adjusted from the visible to the infrared region by adjusting the morphologies and sizes of the nanoparticles. However, noble metals have several disadvantages, including high cost and poor stability. This has prompted the search for low-cost, non-noble-metal-based alternative plasmonic nanomaterials.

The increasingly severe global climate concern and the excessive reliance on fossil fuels demand new energy collection and utilization means such as solar energy harvesting. Traditional Au and Ag plasmonic nanomaterials have proven to be able to enhance the performance of solar energy-harvesting materials and devices because of their extraordinary light-capturing abilities. Efficient nanoscale light absorbers working in a wide spectral range are strongly desired for these applications. Au and Ag plasmonic nanoparticles have strong but narrow absorption peaks that are typically in the visible and near-infrared regions. However, a significant portion of the solar spectrum falls in the near-infrared region, with ∼37% of the solar energy being in the spectral range above 800 nm. In this regard, it is of paramount importance to develop alternative plasmonic nanomaterials that support strong LSPRs over a large portion of the solar spectrum. Several types of alternative plasmonic materials have been proposed, including metal nitrides, metal oxynitrides, heavily doped metal oxides, and silicides.^10^ Among these alternative plasmonic materials, transition metal nitrides, especially TiN, are excellent materials for solar energy harvesting applications.^11^ TiN nanomaterials exhibit broadband LSPRs, which allow them to absorb visible and near-infrared light.^12^ In addition, they are cheaper than noble metals. They have outstanding mechanical and chemical stability, which makes them suitable for harsh-environment applications.^13^ In fact, TiN nanoparticles have proven to be the top alternative to Au ones regarding light-to-heat conversion.^11,14,15^ As a result, an increasing number of applications based on the photothermal properties of TiN nanoparticles have been demonstrated, including local heat generation,^16^ photothermal catalysis,^17^ self-regulating smart widows,^18^ photothermal therapy,^19^ and seawater desalination.^20^

As freshwater accounts for only 2.5% of all water resources on Earth, the water-shortage crisis has become a serious global challenge. Although heat-based technologies such as multistage distillation and vapor compression have been well established, they often require large infrastructures that are energy-intensive and unavailable in remote or poorly developed areas.^21^ A green, feasible, and economical seawater desalination technique is highly desired. Solar seawater desalination has recently attracted much attention because of its low cost, low energy consumption, and environmental friendliness. One of the challenges for solar seawater desalination is its low output capacity, which severely hinders its practical use. TiN nanomaterials as superb light absorbers are believed to have the potential to solve the problem of the low output capacity. Many research works have demonstrated the control of the size and shape of conventional noble metal nanoparticles to satisfy different applications.^1,9,22^ In contrast, there have still existed great challenges for the preparation of plasmonic TiN nanoparticles with controllable sizes and shapes.^23^ Different deposition methods have been employed to prepare TiN nanostructures, such as chemical vapor deposition,^24^ atomic layer deposition,^25^ physical vapor deposition,^26^ and microwave-plasma-assisted deposition.^27^ The produced TiN nanostructures are limited in amount and are often attached firmly on substrates. Chemically synthesized TiN nanoparticles are free-standing and can be readily integrated with other materials to achieve the desired functions. There have been so far only a few works on the chemical synthesis of TiN nanoparticles.^23,28−30^ However, the lack of size and morphology control of TiN nanoparticles has severely limited the synthetically adjustable spectral range of their LSPRs. It is therefore highly desired to further develop chemical methods for the synthesis of TiN nanoparticles with controllable morphologies and sizes.

In this work, we developed a chemical approach for the synthesis of colloidal TiN nanoparticles with diverse shapes and sizes. The synthesized TiN products include nanospheres (NSs), nanobipyramids (NBPs), and nanorod arrays (NRAs). Such TiN nanoparticles exhibit broad LSPRs, which are particularly attractive for solar energy harvesting applications. The colloidal TiN NSs and NBPs are mixed together and encapsulated into highly porous poly(vinyl alcohol) films for solar seawater desalination. The seawater evaporation rates of the composite films reach 3.8 kg m^–2^ h^–1^, which is the highest among all plasmonic solar seawater desalination systems reported so far. The composite films also have the ability to remove organic contaminants during solar seawater desalination. The high removal percentage of organic contaminants originates from the joint action of the high photocatalytic ability and the superhydrophilicity of the porous TiN-based composite film.

## 2 Results and Discussion

Nanoscale TiO_2_ is one of the most popular photocatalysts.^31^ Many chemical methods have been developed for the synthesis of TiO_2_ nanoparticles with various morphologies such as NSs,^32^ NBPs,^33^ hollow nanostructures,^34^ and nanosheets.^35^ Although the preparation of TiN nanostructures through the nitridation of TiO_2_ nanostructures have been reported before,^29,36−38^ the control of the morphologies and sizes of TiN nanoparticles through the nitridation of TiO_2_ with well-controlled morphologies has not been demonstrated. The preparation of porous TiN nanoshells using a three-step method has recently been reported. The method is complicated and only suitable for spherical TiN nanoshells.^39^ In this work, we handily prepared size-variable TiO_2_ nanoparticles with three different shapes. The morphologies of the obtained TiN products are similar to those of TiO_2_ nanoparticles before nitridation.

**Figure 1:**
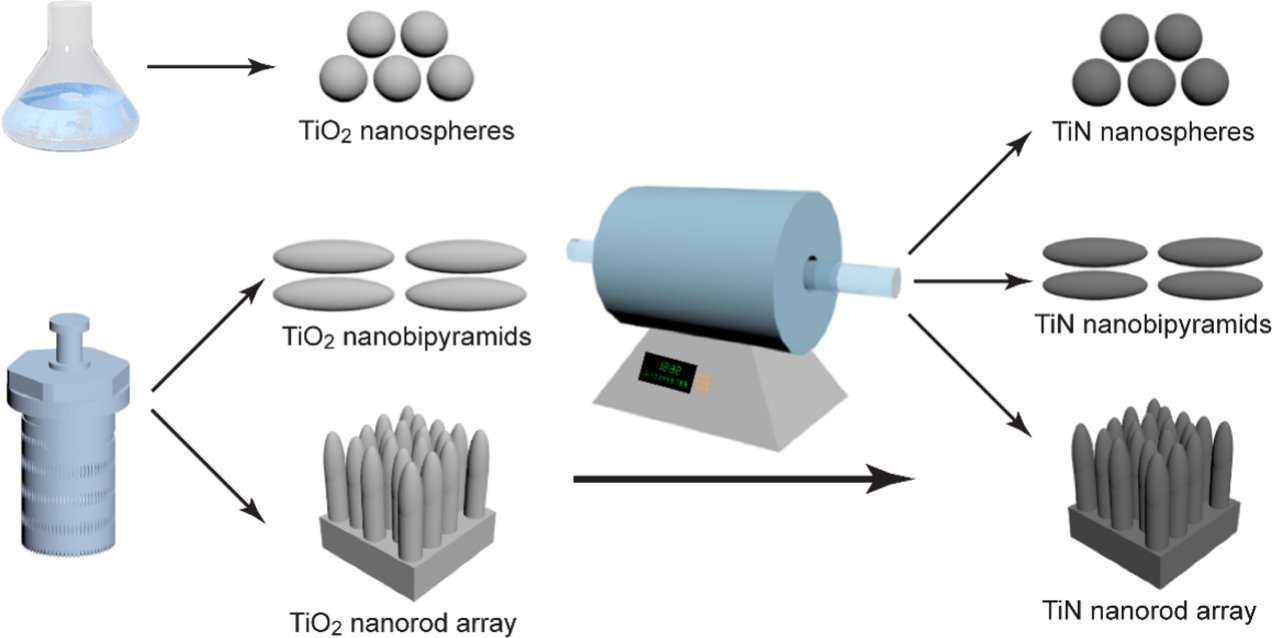
Schematics illustrating the preparation of colloidal plasmonic TiN nanoparticles. The preparation process consists of two steps: the synthesis of TiO_2_ nanoparticles and the post-treatment.

Colloidal plasmonic TiN nanoparticles were prepared in two steps, as schematically illustrated in Figure 1. First, TiO_2_ nanoparticles were prepared. They are TiO_2_ NSs, TiO_2_ NBPs, and TiO_2_ NRAs. The TiO_2_ NSs were prepared by heating the precursor solution in a container, while the TiO_2_ NBPs and TiO_2_ NRAs were prepared hydrothermally. The TiN nanoparticles were then produced through nitridation treatment at a high temperature with an ammonia gas flow.

The uniform spherical morphology of the as-prepared TiO_2_ NSs with their diameters ranging from ∼50 to ∼500 nm is revealed in Figures S1–S3. Both the scanning electron microscopy (SEM) and transmission electron microscopy (TEM) images show that all of the TiO_2_ NS samples are highly uniform. X-ray photoelectron spectroscopy (XPS) indicates that the binding energy of Ti 2p_3/2_ is 458.4 eV and that of O 1s is 529.9 eV (Figure S4), which verifies that the synthesized NSs are TiO_2_. Two methods were used for nitridation treatment. The first method was to directly nitridize the as-synthesized amorphous TiO_2_ NSs. For the second method, the as-synthesized amorphous TiO_2_ NSs were first calcined to give anatase TiO_2_ NSs, which were subsequently nitridized. The SEM images (Figure S5A) of the TiN NSs produced by the two methods show no clear difference in the surface morphology. The X-ray diffraction (XRD) patterns of the two TiO_2_ NS samples before and after nitridation are shown in Figure S5B,C. The diffraction peaks of the TiN NS sample produced through nitridation of the amorphous TiO_2_ NSs are slightly shifted to the larger-angle side compared to those in the standard XRD pattern of TiN, suggesting that the interplanar spacing is slightly reduced. In comparison, the diffraction peaks of the TiN NS sample obtained through nitridation of the anatase TiO_2_ NSs are shifted to the larger-angle side to a larger extent. The possible reason is that the mass density of amorphous TiO_2_ (3.6 g cm^–3^) is smaller than that of anatase TiO_2_ (3.9 g cm^–3^). During the nitridation process, the TiO_2_ sample with a smaller mass density is easier to be nitridized. To nitridize the TiO_2_ NS sample more thoroughly, we therefore carried out the nitridation process on the amorphous TiO_2_ NSs.

**Figure 2:**
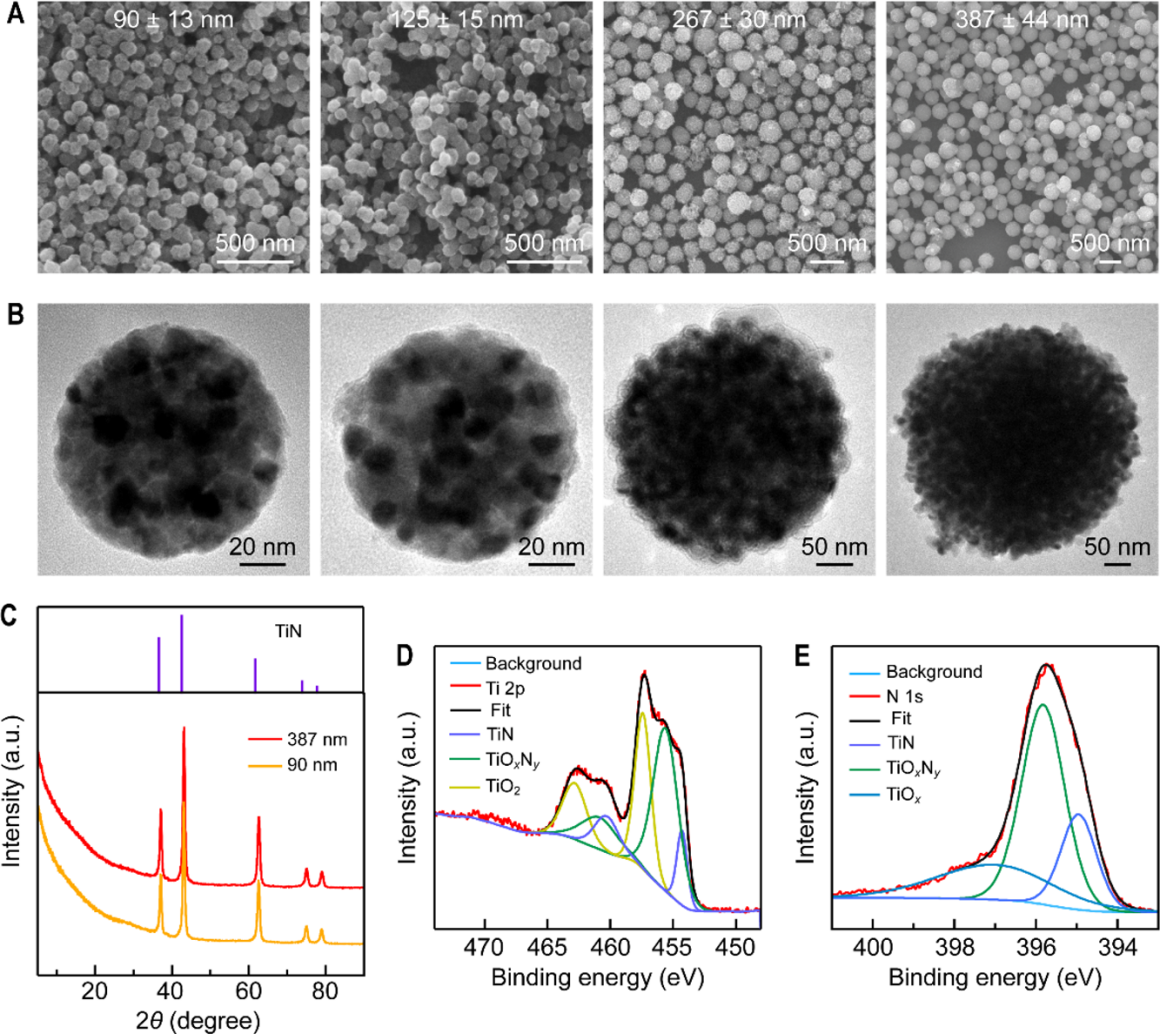
TiN NSs. (A) SEM images of the TiN NSs with four different average diameters. (B) Representative TEM images of the TiN NSs from the four samples shown in (A). (C) XRD patterns of two differently sized TiN NS samples. The diffraction peaks can be indexed according to the standard XRD pattern (JCPDS 87-0628) of TiN. (D) XPS spectra of Ti 2p and fitted peaks. (E) XPS spectra of N 1s and fitted peaks.

The ammonia flow rate is one of the important factors during nitridation treatment. Three ammonia flow rates (0.01, 0.03, and 0.1 L min^–1^) were tested to examine their effect on the nitridation of the TiO_2_ NSs. When the ammonia flow rate is increased, the grain size on the surface of the sample gradually increases (Figure S6A) while the O content in TiN gradually decreases (Figure S6B). The flow rate of 0.1 L min^–1^ was therefore selected to maximize the nitridation extent. The SEM and TEM images show that all of the TiN NSs are of spherical shape and highly uniform in size (Figure 2A,B and Figure S7). Two representative differently sized TiN NS samples exhibit the same XRD patterns (Figure 2C), which proves that the size of the TiO_2_ NSs does not affect the crystalline phase of the nitridized sample. The XPS spectra (Figure 2D,E) reveal the coexistence of TiN, TiO_*x*_N_*y*_, and TiO_2_. The presence of oxygen in the nitridized samples was corroborated jointly by energy-dispersive X-ray (EDX) and XPS analysis. Nonetheless, the XRD patterns of the TiN NS samples produced through nitridation of both the amorphous and anatase TiO_2_ NS samples do not show any diffraction peaks of TiO_2_ (Figure S5C). Since XPS can only probe the surface region of the sample within a few nanometers, we speculate that a majority of the amorphous TiO_2_ component is existent on the surface of the TiN NSs and that the total amount of TiO_2_ in the TiN NSs is too low to be detected by XRD.

The TiO_2_ NSs decrease in size upon nitridation (Figure S8). The shrinkage ratios of the diameter and volume are 23.7 and 55.6%, respectively. The nitridation-induced size reduction originated from the replacement of two oxygen atoms with one nitrogen atom, while the number of titanium atoms is unchanged. To investigate the stability of the TiN NSs under high-temperature conditions, we treated the TiN NSs with an average diameter of 121 nm at 150 °C in air for different durations (Figure S9). The extinction spectra of the TiN NSs remained unchanged after thermal treatment for different durations up to 1 h. The elemental compositions of the treated samples were revealed by EDX to be similar, which confirms that the TiN NSs have good thermal stability in air. We also selected 400 °C as the treatment temperature to further examine the stability of the samples at higher temperatures (Figure S10A,B). The extinction peak of the TiN NSs red-shifts, and the peak intensity gradually decreases with the treatment time. This is because a thicker layer of TiO_2_ is formed on the surface of the NSs. This oxide layer can also be regarded as a carrier depletion layer, which is on the surface with a largely reduced free carrier concentration. The depletion layer can weaken the LSPR, which thus leads to the decrease in the extinction peak intensity of the sample.^40^ In addition, the depletion layer can also change the dielectric environment surrounding the plasmonic TiN NSs, leading to the red shift of the extinction peak. Moreover, the O content gradually increases with the heating time. In contrast, the N content gradually decreases and the Ti content remains unchanged (Figure S10C). The thermogravimetric analysis (TGA) of the TiN NS sample was also performed to examine the thermal stability. The weight of the sample starts to dramatically increase above 300 °C because of surface oxidation (Figure S11), which also proves that the sample has great thermal stability below 300 °C.

**Figure 3:**
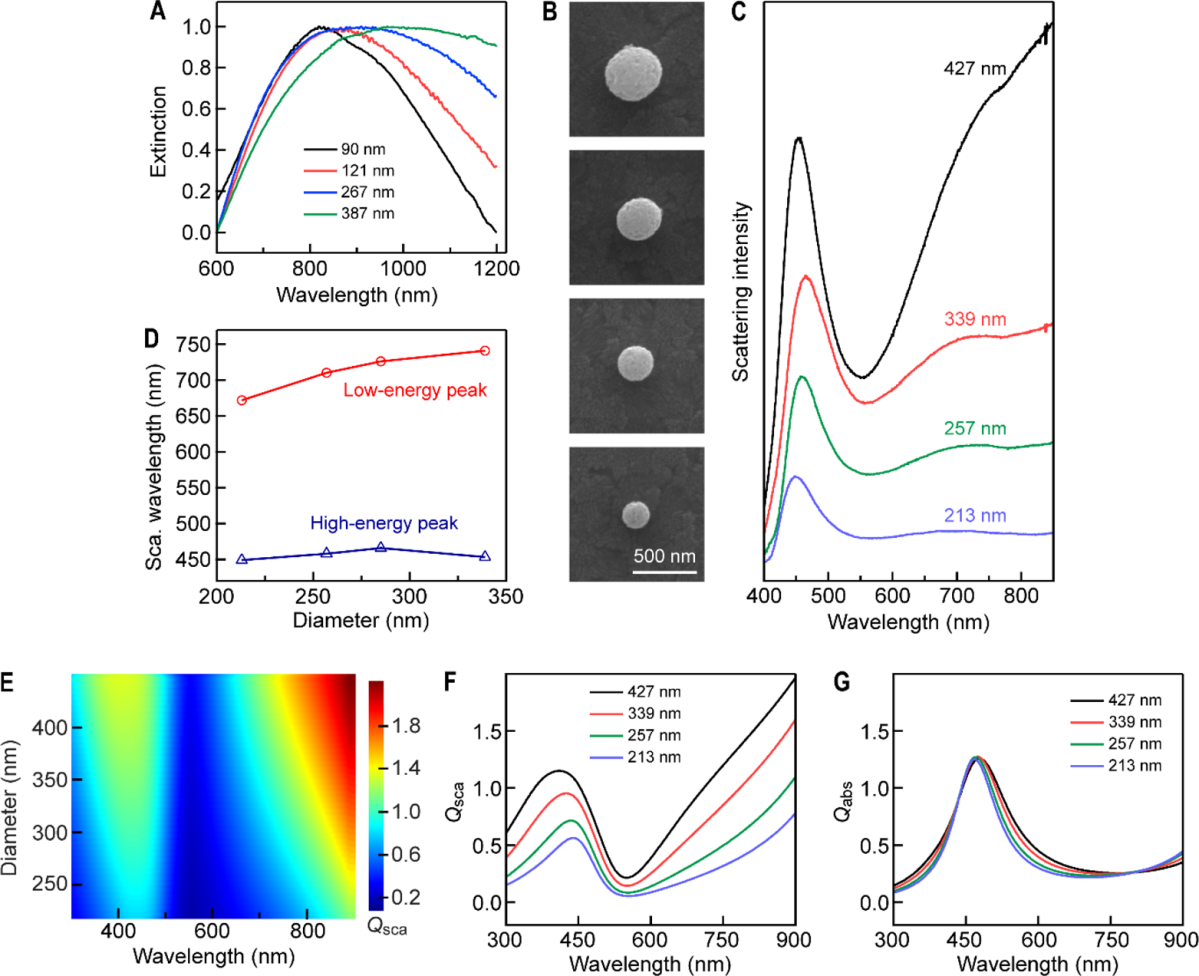
Optical properties of the TiN NSs. (A) Extinction spectra of four differently sized TiN NS samples. (B) SEM images of four representative differently sized TiN NSs. (C) Single-particle dark-field scattering spectra of the TiN NSs. (D) Scattering peak positions as functions of the NS diameter. (E) Calculated scattering efficiency spectra of the TiN NSs with diameters ranging from 250 to 450 nm. (F) Calculated scattering efficiency spectra of four TiN NSs with different diameters. (G) Calculated absorption efficiency spectra of the four TiN NSs.

The extinction spectra of the TiN NSs with four different diameters (90, 121, 267, 387 nm) are shown in Figure 3A. The extinction peak wavelength increases from 818 to 966 nm when the average diameter is increased. The single-particle scattering spectra of four differently sized TiN NSs were measured (Figure 3B,C). There are two prominent scattering peaks for all of the TiN NSs in their scattering spectra. The peak width of the higher-energy peak is narrow, while the peak wavelength changes only slightly as the NS size is varied. In contrast, the lower-energy scattering peak is broad and the peak position red-shifts from 672 to 741 nm as the NS diameter is increased (Figure 3D). The red shift is fundamentally linked to the mechanism of plasmon resonance, a phenomenon arising from the interaction between light and the nearly free electrons within a plasmonic nanoparticle. Reduction in the size of the nanoparticle increases the electromagnetic attraction force between the negatively charged electrons and positively charged nuclei, which in turn increases the plasmon resonance energy and thus causes a blue shift in the plasmon peak. Conversely, the enlargement of the nanoparticle reduces the attraction force and leads to a red shift in the plasmon peak. We subsequently conducted the measurements of the extinction spectra of four TiN NS samples of different sizes, covering a spectral range from 260 to 1300 nm (Figure S12). The TiN NS samples exhibit distinct extinction peaks in the near-ultraviolet and near-infrared regions, which are consistent with the results obtained from the single-particle scattering measurements.

Mie theory calculation of the scattering spectra was performed using the Drude–Lorentz model for the differently sized TiN NSs (Figure 3E). The scattering intensity of the TiN NSs in the near-infrared region becomes stronger as the particle gets larger. The calculated scattering efficiencies of the TiN NSs, as well as the relative contributions from the electric and magnetic resonance modes of different orders, are shown in Figure 3F and Figure S13. For the TiN NSs with small diameters, such as 213 and 257 nm, the magnetic dipole mode is dominant. For the TiN NSs with increasing diameters, the intensities of the magnetic quadruple and electric dipole modes grow more quickly than that of the magnetic dipole mode on the shorter-wavelength side. The calculated single-particle absorption efficiency spectra of the TiN NSs only show a sharp peak at ∼500 nm (Figure 3G), indicating that the extinction signals of the TiN NS samples in the lower-energy region are solely originated from scattering. Taken together, the single-particle scattering spectra calculated based on the Mie theory show good agreement with the measured ones.

The dielectric function of TiN based on the Drude–Lorentz model is shown in Figure S14. The LSPR in TiN can occur in the spectral range longer than 670 nm, where the real part of the dielectric function is negative and the imaginary part is relatively small. As a result, the peak at ∼450 nm for the TiN NSs should originate from interband transitions. This high-energy peak was modeled with a Lorentz oscillator in the Mie-theory-based calculations.

We also calculated the scattering efficiency spectra of the differently sized TiN NSs using the Drude model. The results show good agreement with the measured spectra in the low-energy range (>500 nm, Figure S15). However, the high-energy peak that was observed in the measured scattering spectra (Figure 3C) is missing. The Drude model can be improved by introducing a Lorentz oscillator to obtain a better-matched modeling. The results calculated from the Drude–Lorentz model (Figure 3F and Figure S13) are in significantly better agreement with the measured spectra than those based solely on the Drude model. The strong plasmon resonance in the near-infrared region is enabled by the large carrier concentration in the TiN NSs. In addition, the maximal electric field enhancement of the TiN NSs reaches ∼32 (Figure S16).

Although the plasmon wavelength of the TiN NSs can be tailored in the near-infrared region, it is difficult to further adjust their plasmon wavelength to be longer than 1000 nm. The plasmon wavelength of Au NBPs has recently been reported to exhibit high plasmon wavelength tunability in the near-infrared region, which is highly desired for many applications.^41^ Furthermore, Au NBPs possess two sharp tips that serve as hotspots for large local electric field enhancement. Au NBPs therefore have great potential for photonic, biotechnological, and spectroscopic applications.^41^ Since TiN is a good alternative for Au, it is particularly important to fill the gap in the preparation and optical properties of TiN NBPs. TiN NBPs with different sizes were therefore prepared, and their optical properties were studied. To this goal, TiO_2_ NBPs with different sizes were first prepared (Figure S17). The XRD peaks of the TiO_2_ NBPs show the anatase TiO_2_ phase (Figure S18). The binding energy of Ti 2p_3/2_ for Ti^4+^ is 458.4 eV and the binding energy of O 1s is 529.5 eV (Figure S19), which are consistent with the typical binding energies of TiO_2_. Good size uniformity was verified by TEM images of the TiO_2_ NBPs with different sizes (Figure S20). In order to examine the effect of the ammonia flow rate, we tested the nitridation effect on the TiO_2_ NBP samples with two different sizes (length *L*: 348 ± 41 nm, waist width *W*: 125 ± 15 nm; *L*: 274 ± 20 nm, *W*: 97 ± 7 nm, Figure S21). The morphologies of both samples remain intact after the nitridation treatment. The larger TiN NBPs do not show any plasmon peaks, while the smaller TiN NBPs exhibit a clear plasmon peak in the visible to near-infrared regions. The XRD patterns show that the larger TiN NBPs exhibit the diffraction peaks of both the TiN phase and the anatase TiO_2_ phase and that the smaller TiN NBPs present only the diffraction peaks of the pure TiN phase. This result reveals that the nitridation of the larger TiN NBPs is incomplete at an ammonia flow rate of 0.03 L min^–1^. The ammonia flow rate of 0.1 L min^–1^ was therefore used for the nitridation treatment of the TiO_2_ NBP samples.

**Figure 4:**
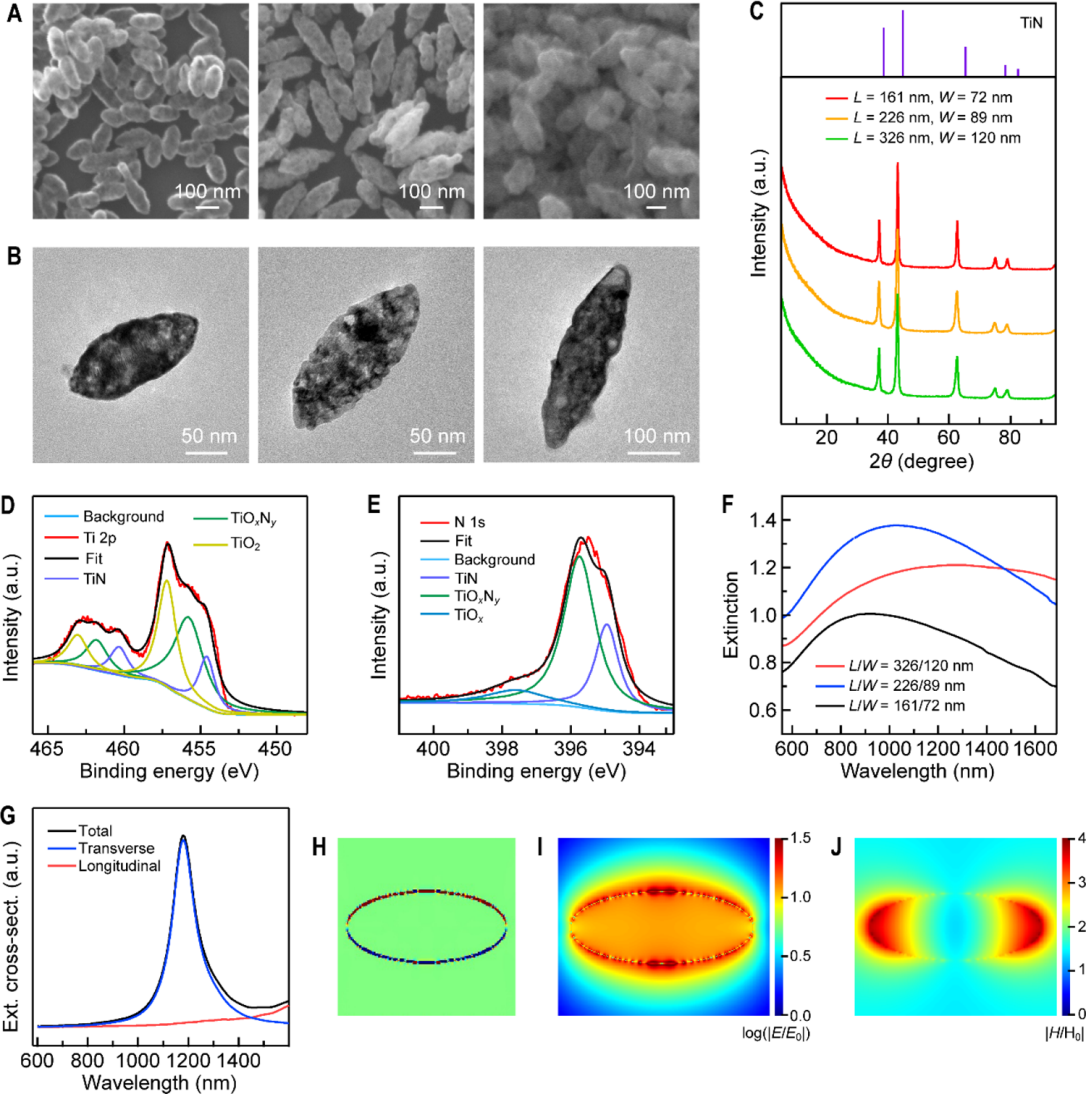
TiN NBPs. (A) SEM images of three differently sized TiN NBP samples. (B) TEM images of the representative TiN NBPs from the three samples. (C) XRD patterns of the three TiN NBP samples. The diffraction peaks can be indexed according to the standard XRD pattern (JCPDS No. 87-0628) of TiN. (D,E) Measured and fitted XPS spectra of Ti 2p (D) and N 1s (E) of the TiN NBPs. (F) Extinction spectra of the three TiN NBP samples dispersed in D_2_O. (G) Simulated extinction cross-sectional spectra of a representative TiN NBP (*L* = 161 nm, *W* = 72 nm). (H–J) Simulated charge distribution (H), electric field enhancement (I), and magnetic field enhancement (J) contours under transverse excitation at the extinction peak in (G).

TiN NBPs with three different sizes were prepared to demonstrate the generality of our nitridation method (Figure 4A,B). The XRD patterns (Figure 4C) of the obtained TiN NBPs show the same TiN phase. The TiN NSs and NBPs all exhibit the characteristic diffraction peaks of TiN (Figure S22A). This result proves that the nitridation conditions are equally applicable for both TiO_2_ NSs and TiO_2_ NBPs. The EDX spectra of the TiN NBPs reveal the existence of a non-negligible amount of oxygen (Figure S22B,C). The extent of nitridation of the TiN NBPs is 75.6 ± 11.6%. The origin of oxygen can be ascribed to the coexistence of TiN, TiO_*x*_N_*y*_, and TiO_2_, as verified by the XPS measurements of the valence states of Ti and N (Figure 4D,E). The extinction peaks of the three TiN NBP samples with different sizes (*L*: 161 ± 13 nm, *W*: 72 ± 4 nm; *L*: 226 ± 16 nm, *W*: 89 ± 7 nm; *L*: 326 ± 24 nm, *W*: 120 ± 5 nm) red-shift as the TiN NBPs become larger (Figure 4F and Figure S23). Finite-difference time-domain (FDTD) simulations were performed to examine the plasmonic properties of the nonspherically shaped TiN NBPs, with the employed model shown in Figure S24A. The extinction in the measured spectral region is dominated by the transverse plasmon resonance mode, as shown by the simulated extinction cross-sectional spectra of the differently sized TiN NBPs (Figure 4G and Figure S24B,D). Figure S25 shows the relative contributions of the scattering and absorption to the extinction of the TiN NBPs. As the TiN NBP becomes larger, the scattering contributes more to the extinction. With the increase in size, the ratios of the scattering cross section to the absorption cross section are 0.23, 0.48, and 1.03, respectively. Compared with the simulation results, the experimental plasmon resonance peaks (Figure 4F) of the TiN NBP samples, however, exhibit much larger red shifts as the sizes are increased. The possible reasons include the surface roughness of the TiN NBPs, size distributions, and the employed dielectric function. To ascertain the possible effect of the surface roughness of the TiN NBPs, we used focused ion beam to cut the TiN NBPs and observed that the interior of the TiN NBPs is completely solid without any porous structure (Figure S26A,B). We therefore considered only the roughness of the surface of the TiN NBPs in the simulations and modified the surface of a TiN NBP to possess both concave and convex defects. The concave defects were randomly positioned air spheres embedded partially in the TiN NBP. The convex defects were randomly positioned TiN NSs attached on the surface of the TiN NBP (Figure S26C). In comparison with the case of the smooth TiN NBP, the transverse plasmon peak in the simulated extinction spectrum of the rough TiN NBP exhibits a red shift of 3.9 nm, with the full width at half-maximum increased by 5 nm (Figure S26D). The effect of the surface roughness can, therefore, be excluded. The discrepancy between the measured and simulated transverse plasmon peaks most probably arises from the size distributions of the TiN NBPs and the employed dielectric function. The latter was obtained from the Drude–Lorentz model for the TiN NSs, and the plasmon resonance peaks of the TiN NSs and NBPs are in different spectral regions. Since the transverse plasmon mode is dominant for the TiN NBPs, we also simulated the charge distribution and electromagnetic field enhancement contours under transverse excitation. The transverse plasmon mode of the TiN NBP (*L*: 161 nm, *W*: 72 nm) shows a dipole nature (Figure 4H). The maximal local electric/magnetic field enhancement reaches ∼32/4 (Figure 4I,J). The simulated charge distribution and electric and magnetic field enhancement contours for the NBPs with the other two sizes give similar results (Figure S27).

We not only synthesized the TiN nanoparticles with controllable morphologies and sizes but also prepared them at the gram scale by our method (Figure S28). The TiN nanoparticles obtained from the nitridation treatment no longer exhibit semiconductor characteristics as the inherent electrical and optical properties are largely changed. The high charge carrier density (2.16 × 10^22^ cm^–3^) in the TiN NBPs was verified through Mott–Schottky measurements (Figure S29). The charge carrier concentration in the TiN NBPs is significantly higher than those of common semiconductors,^41−44^ such as ZnO, TiO_2_, and Cu_2_O. Such a high carrier concentration, which is comparable to those in Au and Ag, directly endows the TiN NBPs with the plasmonic nature that supports a strong light-capturing ability. To further evaluate the plasmonic properties of our low-cost, non-noble-metal TiN NBPs, we compared the similarities and differences in the optical properties between the TiN and Au NBPs. The scattering and extinction cross-section spectra of three Au NBPs with the same sizes as those of the TiN NBPs described above were simulated. Longitudinal excitation was considered because the longitudinal plasmon resonance is dominant and sensitive to the NBP sizes and the wavelength of the weak transverse plasmon mode is nearly independent of the NBP sizes.^45^ The longitudinal dipole plasmon peaks of the Au NBPs were found to red-shift as the particle sizes are increased (Figure S30). The degree of the red shift for the longitudinal dipole plasmon peaks of the Au NBPs is similar to that for the transverse dipole plasmon peaks of the TiN NBPs, but the peaks of the Au NBPs are much narrower, which limits the utilization of the broadband solar energy. The charge distribution contours of the largest Au NBP at the plasmon peaks were also simulated to illustrate the characteristics of each plasmon mode (Figure S31). Compared with the TiN NBP of the same sizes, the Au NBP shows richer plasmon modes.

**Figure 5:**
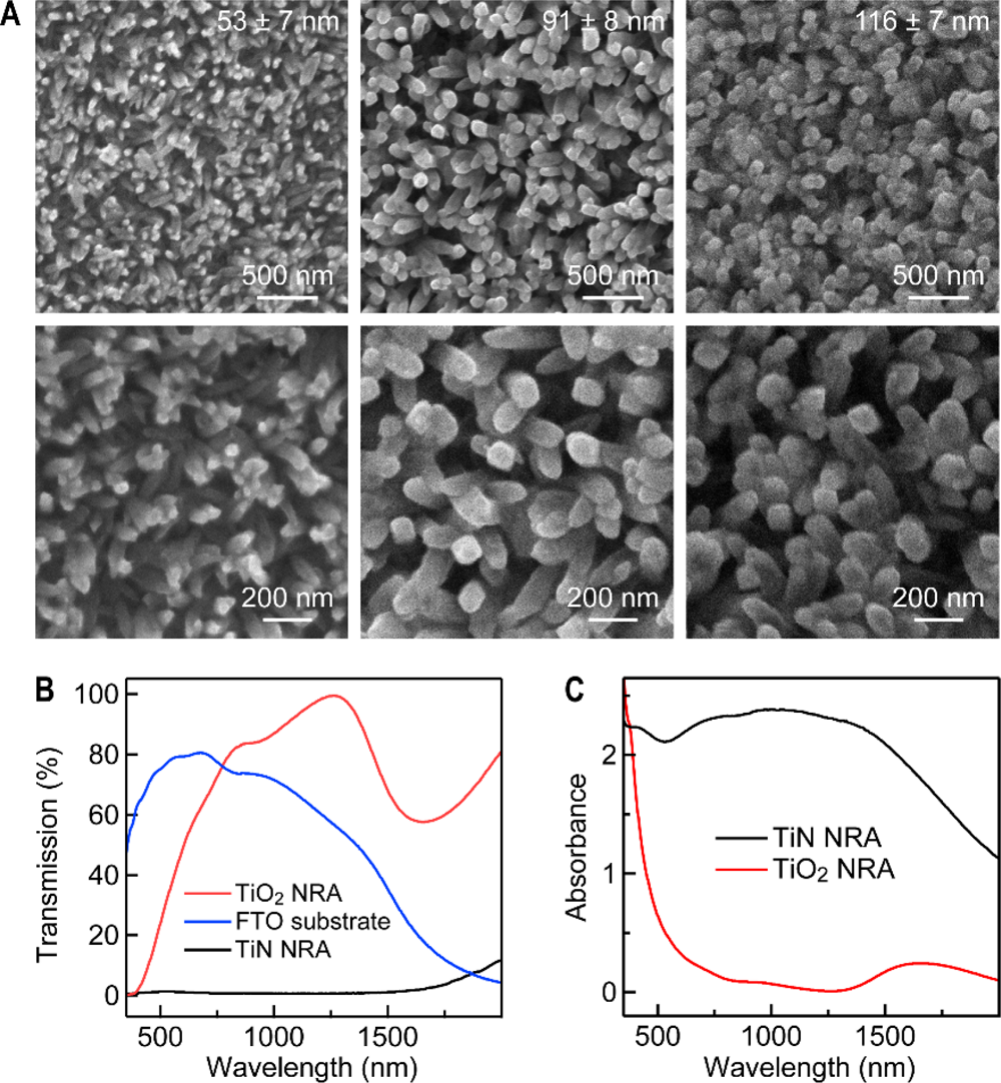
TiN NRAs. (A) SEM images of TiN NRAs with three different diameters. In the bottom row are the SEM images at high magnifications. (B) Transmission spectra of the FTO substrate, a TiO_2_ NRA, and a TiN NRA. (C) Absorbance spectra of TiO_2_ NRA and TiN NRA.

Perfect light absorbers have been explored for decades. They have been used in many solar-harvesting applications, such as photovoltaics,^46^ sensing,^47^ radiative cooling,^48^ and thermal light sources.^49^ In addition to strong light absorption, light-absorbing nanoparticles used to collect solar energy must also be distributed uniformly on the substrates over large areas. We therefore designed a TiN NRA structure as an outstanding light absorber. For the preparation of the TiN NRAs, TiO_2_ NRAs with different sizes were first grown on the conductive surface of fluorine-doped tin oxide (FTO) substrates hydrothermally. TiN NRAs with three diameters (53 ± 7, 91 ± 8, and 116 ± 7 nm) were then produced through the nitridation treatment of the TiO_2_ NRAs (Figure 5A). Their lengths are in the range of 200–400 nm. The transmission spectra of the blank FTO substrate, a TiO_2_ NRA, and a TiN NRA, the latter two of which were deposited on the FTO substrates, were measured. The TiO_2_ NRA shows high transmission in the visible and near-infrared regions, while the TiN NRA hardly transmits light in the wavelength range below 2000 nm (Figure 5B). The absorption measurements show that TiN NRA exhibits an excellent light absorption of more than 90% in the wavelength range from 250 to 2000 nm (Figure 5C). The excellent light absorption ability of the TiN NRAs endows them with a nearly black appearance (Figure S32). Interestingly, the TiN NRAs can be ultrasonicated in aqueous solutions for ∼30 min to give self-supporting sheetlike TiN NRAs (Figure S33). The single-layer TiN NRA sheet structure with a thickness of ∼390 nm remains structurally stable even after long-term ultrasonication.

**Figure 6:**
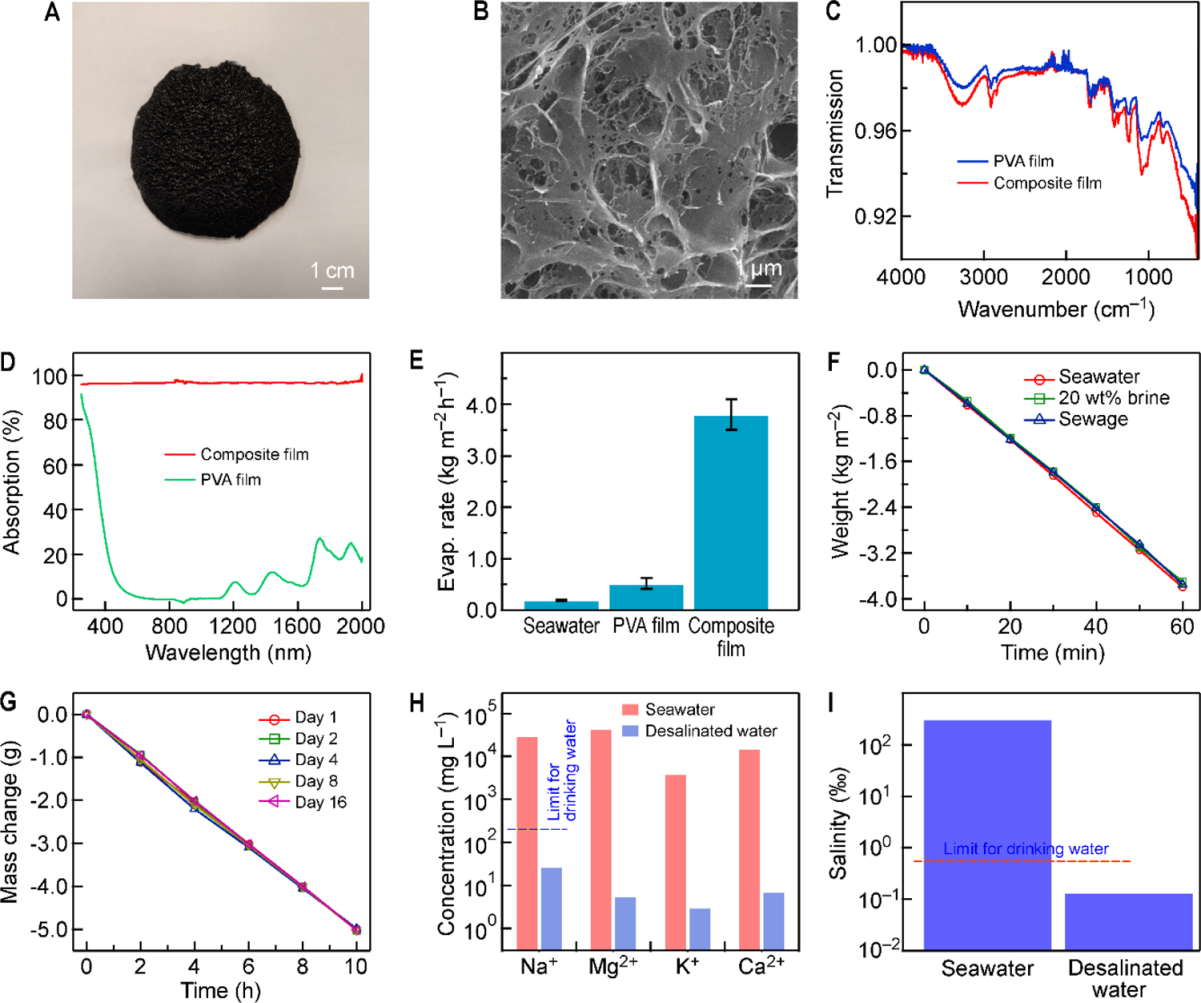
Composite film for solar seawater desalination. (A) Photograph of the TiN NSs/TiN NBPs/PVA composite film. (B) SEM image of the composite film. (C) FTIR spectra of the PVA film and the composite film. (D) Light absorption spectra of the PVA film and the composite film. (E) Solar vapor generation performances of sole seawater, a pure PVA film, and the composite film. (F) Solar vapor generation performances of the composite film in seawater, 20 wt % brine, and the sewage. (G) Solar vapor generation performances of the composite film on the first, second, fourth, eighth, and 16th days. (H) Concentrations of the four common types of metal cations in the seawater and desalinated water. The dashed line represents the WHO standard for Na^+^ in drinking water. (I) Salinity levels of seawater and desalinated water. The dashed line represents the WHO salinity standard for drinking water.

Solar-to-heat conversion offers a new approach to the green and effective production of clean and drinkable water from seawater desalination. Harvesting solar energy and converting it to heat for water evaporation by plasmonic nanoparticles are attractive strategies for achieving this goal. The use of plasmonic nanoparticles can lead to very high light-to-heat conversion efficiencies.^50^ Plasmonic nanoparticles also ensure high surface-area-to-volume ratios for light-to-heat conversion and heat transfer. To judiciously design a plasmonic material with broadband light absorption over the entire solar spectrum, we combined plasmonic TiN NSs and NBPs together as light absorbers. The TiN NRAs were excluded, because their mass production is limited by the preparation method. The selected TiN nanoparticles were further integrated with the poly(vinyl alcohol) (PVA) polymer, which is floatable. The TiN NSs/TiN NBPs/PVA composite films display a black appearance in contrast to the white color of the pure PVA film (Figure S34). The TiN nanoparticles were loaded inside the polymer network. The prepared composite film was cut into a round shape (Figure 6A). The diameter of the composite film is ∼8.5 cm, and the entire surface is completely black, demonstrating strong light absorption in the entire visible range. The TiN nanoparticles are tightly attached to the PVA network (Figure 6B), where the particle attachment is believed to be caused by molecular interactions. Since there are many interparticle gaps among the embedded closely packed TiN nanoparticles within the polymeric matrix, numerous electromagnetic hotspots are generated such that local heat generated near the hotspots can accelerate the evaporation of seawater near the surface of the nanoparticles. Hollow channels with a spacing of 9 mm and a diameter of 2.5 mm were fabricated on the film. They penetrated the film. The transport of the salt precipitated on the film surface can be enhanced through the hollow channels.^51^

The Fourier-transform infrared (FTIR) spectra show that the TiN NSs/TiN NBPs/PVA composite film has six functional groups, which are O–H, C–H, C–O, C=O, C–C, and Ti–N groups (Figure 6C). The absorption peaks observed at the wavenumbers of 3267.01, 2914.78, 1707.12, 1373.52, 1243.07, 823.74, and 600 cm^–1^ are indicative of the presence of chemical functional groups. They can be ascribed to the stretching vibrations of the O–H, C–H, C=O, and C–O bonds, the bending of the C–H bonds, and the stretching vibrations of the C–C and Ti–N bonds, respectively. The C–H and C=O groups are formed through glutaraldehyde cross-linking. The highly porous structure of the PVA framework also endows the film with flexibility and tenacity. The composite film can quickly recover to its original thickness after the pressing force from a tester’s finger was removed (Movie S1). In addition, the film can easily float on the water surface (Movie S2). Enthalpy recycling and taking energy from the environment are two effective approaches to exceed the theoretical solar seawater evaporation limit.^52,53^ However, the reason for the TiN composite film exhibiting a high solar seawater evaporation performance is based on the following synergistic effect. The film can greatly facilitate water evaporation due to the reduced evaporation enthalpy of water in the hydrogel network.^54^ Water molecules that interact strongly with the functional groups on the hydrogel backbone are called bound water. There is also intermediate water, which is weakly bound to the polymer chain and located between the bound water and the free water (bulk water). The intermediate water requires less energy to escape from surrounding water molecules.^54,55^ The intermediate water has been found to evaporate at a rate 86 times faster than the free water.^56^ On the other hand, there exists a thermal barrier at the interface of the plasmonic nanoparticles and water during desalination. Heat is difficult to transfer from the nanoparticle to the surrounding water because steam is a poor heat conductor. The steam shell will gradually increase in thickness and finally escape to the water surface in the desalination process because of the buoyancy force. As a result, there is no need to heat the entire water volume to the boiling point.^57^ The water vapor generated inside the composite film is divided by the porous structure into small parts, which greatly reduces the thermal convection. The porous structure inside the film can also provide channels for the transfer of water and the escape of steam. In addition, the complex interplay between light scattering and absorption is of high significance. The interplay originates from the porous and interlocking structure of the PVA membrane together with the aggregation effect of the TiN nanoparticles. The incident light undergoes multiple scattering events in the PVA network. A large portion of the scattered light is reabsorbed by the TiN nanoparticles, which benefits the maximal utilization of the incident light. The reabsorption process therefore further enhances the energy harvesting efficiency and the energy conversion efficiency of the composite film for solar seawater desalination.

The TiN NSs/TiN NBPs/PVA composite film has an extremely high light absorption ability of ∼96.5% in the visible and near-infrared regions compared with that of the PVA film (Figure 6D). To examine the seawater desalination performance, a 50 mL beaker was filled with seawater, with the surface of the seawater covered with our TiN NSs/TiN NBPs/PVA composite film. The composite film was placed on top of a thin and light packaging foam, which functioned as a thermal insulating layer to prohibit heat loss to the bulk seawater below. In addition, because of its low density, the packaging foam can also provide extra buoyancy to make the composite film float on the seawater surface. The PVA polymer loaded with the TiN nanoparticles can also function as a water-wicking material to continuously absorb and transport seawater around the TiN nanoparticles. The hydrophilicity of the PVA film and the TiN composite film were determined by the water contact angle test. The results show that the contact angle of the PVA film is 62° while that of the TiN composite film is almost 0°. The loading of the TiN nanoparticles into the PVA film therefore renders the composite film superhydrophilic (Figure S35, Movies S3 and S4). The localized heat generated on the surface of the TiN nanoparticles illuminated by light evaporates seawater, thereby accelerating the process of solar seawater desalination. The superhydrophilic composite TiN film also helps water molecules more easily reach the surface of the TiN nanoparticles, thereby further increasing the evaporation rate of seawater. To directly visualize the excellent light-to-heat conversion performance of the composite film, an infrared thermal imager was used to record the temperature changes of the film under different illumination times (Figure S36). As the illumination time was increased, the surface temperature of the film increased from ∼20 to ∼44 °C, which confirms the excellent light-to-heat conversion performance of the composite film. To measure the solar seawater desalination rate, a xenon lamp was combined with an AM 1.5 solar filter to simulate sunlight. The light intensity of the solar simulator was set to 1000 W m^–2^. The solar water generation rate was measured every 10 min. Compared with the control groups of sole seawater and the pure PVA film, the seawater desalination performance of the composite film is significantly improved. A water evaporation rate of 3.8 ± 0.3 kg m^–2^ h^–1^ was achieved (Figure 6E and Table S1). The standard deviation of the seawater desalination rate for the TiN composite film was obtained by testing the same TiN composite film five times.

The evaporation enthalpy of seawater in the composite film was measured by differential scanning calorimetry (DSC) to be 1379 J g^–1^, which is much smaller than that of sole seawater (2406 J g^–1^) (Figure S37A).^58^^,^^59^ Since the composite film is completely hydrated during seawater evaporation, the more accurate evaporation enthalpy of the composite film should only include the energy involved in the evaporation of free water and intermediate water. We used a beaker filled solely with seawater and a beaker with a composite film floating on seawater in the same confined space and calculated the mass reduction of the two beakers per unit time. The evaporation enthalpy of the composite film can be calculated using the same input energy as for sole seawater^52^
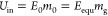
1where *U*_in_, *E*_0_, and *m*_0_ are the identical power input of seawater vaporization, evaporation enthalpy, and mass change of sole seawater, respectively, *m*_g_ is the mass change in the case of the composite film, and *E*_equ_ is the equivalent evaporation enthalpy in the case of the composite film. The calculated evaporation enthalpy of seawater for the composite film is 903 J g^–1^ (Figure S37B). Based on the evaporation enthalpy and evaporation rate of seawater in the composite film, we calculated the energy efficiency η of the collected solar vapor (Figure 6E) during seawater desalination to be 95.3% according to the following equation:
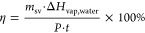
2where *m*_sv_ is the mass of solar vapor (in g), Δ*H*_vap,water_ is the evaporation enthalpy of water (in J/g), *P* is the illumination light power (in W), and *t* is the illumination time (in s).

To further extend the applicability of our composite film, we tested the desalination performances of the composite film in 20 wt % brine and sewage, respectively. The water evaporation performances were found to be consistent with those for sole seawater. These results prove that our composite film can be used to produce freshwater from hypersaline water and sewage (Figure 6F). To ascertain the stability of the TiN NSs/TiN NBPs/PVA composite film during desalination, the composite film was evaluated on the first, second, fourth, eighth, and 16th days. The desalination performances of the composite film are similar among the five tests, which demonstrates the high stability of the composite film (Figure 6G). SEM imaging and XRD were performed on the composite film after the stability test (Figure S38). The results reveal a remarkable similarity in the SEM images and XRD patterns of the composite film before and after the stability test, which further confirms the high stability of the composite film. To evaluate the antifouling performance of our composite film, 1.0 g of NaCl that was equivalent to the amount produced in 1 day by desalinating seawater in 10 h of sunlight was placed on top of the solar steam generation system. After 5 h, the NaCl precipitate dissolved back into seawater (Figure S39). Owing to the good antifouling property of the TiN composite film, the desalination efficiency and stability will not be affected by the salt accumulated on the composite film.

To further demonstrate solar seawater desalination and freshwater collection with our composite film, we fabricated a small-scale and feasible device. Two containers were made of transparent acrylic plates. The top of the large container was open, inclined, and covered with a transparent plastic film. A small container was placed inside the large container, which was filled with seawater. The light-absorbing composite film was positioned on the seawater surface (Figure S40A). When the container was illuminated by the solar simulator, the steam evaporated from the seawater and eventually condensed under the sloped-roof plastic film. The formed water droplets flew into the larger container obliquely along the transparent plastic film (Figure S40B). The enlarged photograph taken during the seawater desalination process shows that a number of water droplets condensed on the inner surface of the plastic film (Figure S40C). Inductively coupled plasma-mass spectrometry (ICP-MS) was employed to quantify the ion concentrations (Na^+^, Mg^2+^, K^+^, Ca^2+^) in evaporated water and check if the evaporated water meets the drinking water standard (Figure S41). The concentrations of all four common types of metal cations in desalinated water were found to meet the drinking water standard stipulated by the World Trade Organization (WTO) (Figure 6H). In addition to the four types of cations commonly found in seawater, we also checked the concentrations of 61 other ions. We found that these ion concentrations all meet drinking water standards (Table S1). In particular, the Ti concentration is only 0.0002 mg L^–1^ in desalinated water, which proves that our TiN nanoparticles do not contaminate desalinated water. Moreover, the salinity and concentration of chloride ions of the desalinated water were also found to meet the drinking water standard (Figure 6I).

A key limitation of current photothermal materials is their inability to separate water from contaminating volatile organic compounds (VOCs), which can be evaporated together with water and cause secondary pollution of desalinated water. VOCs can sometimes even be enriched in desalinated water.^58,60^ To examine the removal rate of organic pollutants in evaporated pure water using our composite film, the composite film was placed in an aqueous phenol solution at a concentration of 100 mg L^–1^ and the phenol content in the evaporated solution upon light illumination was measured. The phenol content in the evaporated water is only 1.7 mg L^–1^ (Figure S42A). The removal percentage of phenol reaches 98.3%. This result shows that the phenol content in the evaporated water meets the drinking water standard.^61^ To check whether the removal of phenol is caused by adsorption and self-decomposition, we further conducted two control experiments. Specifically, the composite film was immersed in an aqueous phenol solution of 100 mg L^–1^ in the dark for 3 h. The residual phenol content was determined to be 98.17 ± 0.02 mg L^–1^. This result indicates that molecular adsorption on the film has only a marginal effect on the removal of phenol. In addition, a similar experiment was carried out under illumination conditions for 3 h. The residual phenol content was found to be 99.55 ± 0.03 mg L^–1^. This result indicates that the photoinduced self-decomposition is negligible for phenol. Our XPS and EDX characterization results described above indicate that there exists a TiO_2_ layer on the surface of the TiN nanoparticles. Moreover, electron paramagnetic resonance (EPR) measurements reveal that the TiN nanoparticles possess a strong symmetric peak with a *g* value of 2.001, which is a characteristic of oxygen vacancies (Figure S42B).^62,63^ Based on the existence of the TiO_2_ layer and oxygen vacancies, we reasoned that our TiN nanoparticles are photocatalytically active.^64^ To confirm this, we checked the residual amount of phenol after the composite film was exposed to light for 3 h in a closed container containing phenol. The residual amount of phenol was found to be 30.21 mg L^–1^. The photocatalytic degradation percentage of phenol is therefore 69.8%. In addition to the photocatalytic degradation of phenol by the TiN composite film, we speculated that the high removal percentage of phenol is also related to the diffusion ability of phenol and water on the surface of the film.^65^ The contact angle of the phenol solution on the TiN composite film was measured to be 47° (Figure S43). The TiN composite film is therefore more likely to adsorb water than phenol. As a result, the high removal percentage of phenol during the evaporation process originates from the joint action of the photocatalytic activity and the higher water diffusion ability of the composite film.

According to the National Academies of Science, Engineering, and Medicine of the United States, men need ∼3.7 L of fluid per day and women need ∼2.7 L of fluid per day.^66^ These fluids include water, other beverages, and fluids in food. Another advantage of the solar water desalination system based on our TiN composite film is that it can provide freshwater for remote areas where large-scale freshwater plants are difficult to be built. Our TiN composite film is required to have an area of 0.13 m^–2^ in order to provide 4 L of freshwater every day, which is enough to meet the daily drinking water need of an adult. The cost of the materials for constructing a solar seawater desalination device with the composite film of that area is estimated to be US$0.75–1.40.

## 3 Conclusions

In summary, this study undertakes a comprehensive investigation of the plasmonic attributes and innovative utilization of differently shaped and sized TiN nanoparticles in solar-driven seawater desalination. Notably, the study reveals a distinct plasmon resonance in the near-infrared region, stemming from a substantial increase in the electron carrier concentration within TiN. The adoption of the Drude–Lorentz model assists in our understanding of the optical properties of TiN nanoparticles. The deep understanding is further facilitated by a synergistic approach combining experimental measurements, theoretical modeling, scattering assessments, and thermal stability investigations, which collectively advance the comprehension of the optical response of TiN nanoparticles. The composite film, amalgamating the TiN nanoparticles within the PVA matrix, showcases remarkable light absorption across a wide spectral range, fostering electromagnetic hotspots for intensified localized heating and seawater evaporation. Notably, the porous architecture of the composite film, augmented by engineered hollow channels, bolsters salt transport and diminishes fouling. The intrinsic hydrophilicity of the film further enhances the water contact and accelerates water evaporation. This work offers substantial contributions to decentralized freshwater generation, particularly pertinent in resource-limited settings, presenting a sustainable solution to water scarcity.

## 4 Methods

### Chemicals

Titanium(IV) oxysulfate (TiOSO_4_), tetramethylammonium hydroxide pentahydrate (HON(CH_3_)_4_·5H_2_O), ethylene glycol (99.8 wt % in water), poly(vinyl alcohol) (PVA, MW 13,000–23,000), titanium(IV) butoxide (97 wt %), and titanium(IV) isopropoxide (Ti[OCH(CH_3_)_2_]_4_) were purchased from Sigma-Aldrich. Polyethylene glycol methacrylate methyl ether (MW 5000) was acquired from Fluka. Aqueous ammonia solution (35 wt %) was obtained from Emsure. Aqueous hydrogen peroxide solution (50 wt %) was purchased from Aladdin. Hydrochloric acid (37 wt % in water) was obtained from RCI Labscan. Absolute ethanol (99.97 wt %) was acquired from VWR Chemicals. Acetone was purchased from Fisher Scientific. Phenol (99.5%) was purchased from Merck KGaA. Deuterium oxide (deuteration degree ≥99.9%) was purchased from Cambridge Isotope Laboratories. Deionized water with a resistivity of 18.2 MΩ cm was used throughout all experiments.

### Synthesis of the TiO_2_ NSs

The TiO_2_ NSs were prepared solvothermally. Typically, titanium(IV) oxysulfate powder (1 g) was dissolved in a conical flask filled with deionized water (120 mL). After the solution was stirred for 10 min, NH_3_·H_2_O (16 mL, 35 wt %) and H_2_O_2_ (8 mL, 50 wt %) were added separately, followed by stirring for 30 min until the solution turned yellow transparent. Absolute ethanol (30 mL) was then added dropwise to the flask. The flask was subsequently kept at 80 °C for 12 h. The growth solution in the flask was finally centrifuged (5000 rpm, 10 min), with the supernatant discarded. The resultant TiO_2_ NSs were dried and stored in a centrifuge tube. The diameter of the TiO_2_ NSs was adjusted by varying the amount of the titanium(IV) oxysulfate powder.

### Synthesis of the TiO_2_ NBPs

The TiO_2_ NBPs were prepared hydrothermally. Typically, tetramethylammonium hydroxide (7.5 mg) and deionized water (30 mL) were mixed and stirred evenly. After the temperature of the solution was raised to 80 °C, ethylene glycol (30 mL) and titanium isopropoxide (1 mL) were added dropwise at 80 °C. The mixture solution was stirred vigorously until a final clear solution was obtained. The reaction mixture was subsequently transferred to a Teflon-lined autoclave and kept at 230 °C for 24 h. The resultant TiO_2_ NBPs were centrifuged and washed with ethanol three times. The sizes of the TiO_2_ NBPs were adjusted by varying the amount of titanium isopropoxide.

### Preparation of the TiO_2_ NRAs

The TiO_2_ NRAs were also prepared hydrothermally. Specifically, concentrated HCl (35 mL, 37 wt % in water) and deionized water (35 mL) were slowly added into a beaker and stirred evenly. Titanium butoxide (0.6 mL) was added to the above solution and stirred for 30 min. The mixture solution was then transferred into a hydrothermal autoclave reactor. The FTO-coated glass slides were cleaned ultrasonically with acetone, ethanol, and deionized water. The conductive side of the FTO-coated glass slide was faced up and placed at the bottom of the autoclave reactor. The reactor was then kept at 150 °C for 2 h. The resultant TiO_2_ NRAs were washed with deionized water three times. The diameter of the TiO_2_ nanorods was adjusted by varying the amount of titanium butoxide.

### Nitridation of the TiO_2_ Nanoparticles

The TiN nanoparticles with different morphologies and sizes were obtained through the nitridation treatment of TiO_2_ nanoparticles (NSs, NBPs, and NRAs) in an ammonia-flowing environment. The ammonia flow rate was 0.1 L min^–1^ except when otherwise mentioned. The heating temperature and time were 800 °C and 5 h, respectively.

### Preparation of the TiN NSs/TiN NBPs/PVA Composite Films

For the preparation of the composite film, PVA (1.125 g) was dissolved into deionized water (15 mL) and stirred evenly, followed by the addition of glutaraldehyde solution (140 μL, 50 wt %). The obtained solution was labeled as solution 1. An aqueous solution composed of the TiN NSs (25 mg), the TiN NBPs (25 mg), and HCl solution (750 μL, 3 wt %) was labeled as solution 2. Solutions 1 and 2 were subsequently mixed uniformly in a beaker and subjected to ultrasonication for 30 min. The obtained composite film was treated with a freeze-dryer machine (FD-1A-50, Tianxiang Instrument Co.) at −60 °C for 12 h.

### Characterization

SEM imaging was performed on a JEOL JSM7800F microscope operated at 20 kV. The elemental compositions of the samples were determined on the same microscope equipped with an Oxford EDX analysis system. TEM imaging was carried out on an FEI Tecnai Spirit microscope operated at 120 kV. XPS was performed on a PerkinElmer PHI 5000C system. XRD patterns were acquired on a Rigaku SmartLab diffractometer equipped with Cu Kα radiation. TGA was carried out on a PerkinElmer TGA 6 thermogravimetric analyzer. The absorption spectra of the sample powders were measured on a PerkinElmer Lambda 950 ultraviolet/visible/near-infrared spectrophotometer using the integrating sphere mode. The infrared thermographies of the temperature evolution of the solar vapor generation system were obtained with a Fluke TiX580 infrared camera. The ICP-MS measurements were performed on an Agilent 7500a ICP-MS system. The EPR spectra were recorded on a JES-FA300 electron spin resonance spectrometer. The water contact angle tests were carried out on a DSA-X ROLL automatic tilting optical contact angle measuring instrument. DSC measurements were performed on a PerkinElmer Pyris 1 DSC, with a heating rate of 5 °C min^–1^ and a temperature range from 25 to 200 °C.

### Solar Seawater Evaporation Measurements

To measure the solar evaporation rate, a xenon lamp with an AM 1.5 solar filter was used to simulate sunlight. A piece of packing foam was placed under the composite film to act as a thermal insulator. It was also used to adjust the position of the composite film relative to the water surface. The seawater was taken from Mirs Bay in Shatin, Hong Kong. The light intensity of the solar simulator was set at 1000 W m^–2^. It was measured using a Kipp & Zonen irradiance meter (CMP3). The seawater evaporation rate was measured every 10 min. The seawater desalination rates of the composite films prepared under the same conditions are similar.

### Phenol Removal and Determination

To investigate the removal percentage of organic pollutants in the evaporated pure water using our composite film, the composite film was placed on an aqueous phenol solution at a concentration of 100 mg L^–1^. The phenol content in evaporated water upon light illumination was determined. To examine the photocatalytic degradation of organic pollutants using our composite film, we determined the residual amount of phenol after the system was exposed to light for 3 h in a closed container containing phenol at a concentration of 100 mg L^–1^. High-performance liquid chromatography was employed to determine the concentration of phenol. A 4.6 × 150 mm SB-C18 column (Agilent) was used, and the mobile phase was a mixture of water and methanol at a volume ratio of 65:35.

### Electrochemical Measurements

The Mott–Schottky plots were obtained by an impedance-potential technique. The interfacial capacitance (*C*) between the working electrode and the electrolyte and the imaginary part of the impedance (*Z*_i_) follows the equation below:
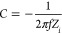
3where *f* (1000 Hz) was the frequency used during the impedance-potential measurement. The flatband potential (*E*_fb_) and apparent carrier concentration (*n*) were obtained according to the following relationship:^67,68^
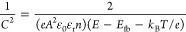
4where *A* is the interface area between the working electrode and the electrolyte, *e* is the elementary electron charge, ε_0_ is the vacuum permittivity, ε_r_ is the relative permittivity of the electrode material at the frequency *f*, *n* is the carrier concentration, *E* is the applied voltage, *k*_B_ is the Boltzmann constant, and *T* is the temperature at the Kelvin scale. The plot of *A*^2^/*C*^2^ against *E* gave a straight line, from which *E*_fb_ was determined from the intercept on the *E* axis. The electron carrier concentration *n* was calculated from the slope of the straight line.

### Single-Particle Dark-Field Scattering Measurements

Single-particle dark-field scattering spectra were recorded on an upright optical microscope (Olympus, BX60) that was integrated with a quartz-tungsten-halogen lamp (100 W), a monochromator (Acton, SpectraPro 2360i), and a charge-coupled device camera (Princeton Instruments, Pixis 400). During the measurements, the camera was thermoelectrically cooled to −70 °C. A dark-field objective (100× , numerical aperture 0.9) was employed for both exciting the individual nanoparticles with unpolarized white light and collecting the scattered light. The scattering spectrum from an individual nanoparticle was corrected by first subtracting the background spectrum taken from the adjacent region without any nanoparticles and then dividing it with the precalibrated response curve of the entire optical system. The exposure time was set to 20 s. A pattern-matching method was employed to locate the same nanoparticles on the dark-field scattering images and the SEM images.^69^

### Electrodynamic Calculations and Simulations

The optical properties of the TiN NSs were calculated according to the Mie theory, as reported in our previous work.^70^ The dielectric function was based on the Drude or Drude–Lorentz model. The refractive index of the surrounding environment (*n*_m_) was set to 1.0 for air. The scattering efficiency of a spherical particle at wavelength λ can be obtained from the following equations:
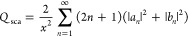
5
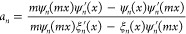
6
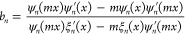
7where *x* = 2π*n*_m_*r*/λ is the size parameter and *m* = *n*/*n*_m_ is the index parameter with *n* being the refractive index of TiN. ψ_*n*_(*t*) = *tj*_*n*_(*t*), where *j*_*n*_(*t*) is the regular Hankel Riccati–Bessel function. ξ_*n*_(*t*) = *th*_*n*_^(1)^(*t*), where *h*_*n*_^(1)^(*t*) is the first-kind Hankel Riccati–Bessel function. All scattering and absorption spectra and relative contributions from the electric and magnetic resonance modes of different orders were calculated using MATLAB. The FDTD simulations were performed using FDTD solution 8.19 (Lumerical). In the simulations, a total-field scattered-field source was launched into a box containing a target nanoparticle. The refractive index of the surrounding medium was set to 1.0 for air. The dielectric function of gold was taken from Johnson and Christy’s experimental data. A mesh size of 2 nm was used for calculating the spectra, while a mesh size of 0.5 nm was employed for calculating the charge distribution and electric/magnetic field enhancement contours of the Au NBP. The Au NBP was modeled with the waist width and length set at 120 and 163 nm, respectively. The tip angle and tip radius were 20° and 3 nm, respectively.

## Supporting Information Available

Flexibility of the composite film (MP4)
Buoyancy of the composite film (MP4)
Contact angle test of the composite film: water droplets on the pure PVA film (MP4)
SEM images; statistics of the diameters of the TiO_2_ NSs; TEM images, and size distributions of the TiN nanoparticles; XPS spectra; nitridation of the TiO_2_ NSs by two different methods; morphology and composition of the TiN NSs; statistics of the diameters of the TiN NSs; shrinkage of the TiN NSs induced by nitridation; thermal treatment of the TiN NSs at 150 °C in air; heat treatment of the TiN NS sample at 400 °C in air; TGA traces; extinction spectra of the four differently sized TiN NS samples in the spectralrange from 260 to 1300 nm; calculated scattering efficiency spectra of the TiN NSs; modeled dielectric function of TiN; calculated scattering efficiency spectra of the TiN NSs; electric field enhancement of the four differently sized TiN NSs; TiO_2_ NBP samples; XRD patterns; XPS spectra of a representative TiO_2_ NBP sample; TEM images of the TiO_2_ NBP samples with different sizes; nitridation of the differently sized TiO_2_ NPBs; characterization of the TiN NBPs; normalized extinction spectra of the differently sized TiN NBP samples; FDTD-simulated extinction spectra of the TiN NBPs; FDTD-simulated absorption and scattering spectra for the three differently sized TiN NBPs; examination of the effect of the surface roughness; simulated charge distribution and electromagnetic field enhancement contours; Gram-scale synthesis of the TiN nanoparticles; Mott–Schottky plot of a representative TiN NBP sample; simulation of the Au NBPs; simulation of the Au NBP; photographs of the NRAs; SEM images of the TiN NRA after ultrasonication; photographs of the films; water contact angle tests; infrared images; evaporation enthalpy of seawater; SEM images and XRD patterns of the TiN/PVA composite film; antifouling performance; seawater desalination device; calibration curves for the ICP-MS measurements; phenol determination and EPR measurement; optical image of a phenol solution droplet on the composite film; solar vapor generation rates and solar energy conversion efficiencies of representative works on solar seawater desalination; concentrations of the ions in the desalinated water (PDF)
Contact angle test of the composite film: water droplets on the TiN NSs/TiN NBPs/PVA composite film (MP4)
