# Supplementary material for: Colloidal Plasmonic TiN Nanoparticles for Efficient Solar Seawater Desalination
Source: ACS Appl Mater Interfaces. 2023 Nov 20;15(48):55856–69. doi: 10.1021/acsami.3c13479 (PMC10711720; doi:10.1021/acsami.3c13479)
Supplement: Supplementary file 4 — am3c13479_si_004.pdf [file am3c13479_si_004.pdf]

Supporting Information

Colloidal Plasmonic TiN Nanoparticles for Efficient Solar Seawater Desalination

Xiaopeng Bai,[1] Shiu Hei Lam,[1] Jingtian Hu,[1] Ka Kit Chui,[1] Xiao-Ming Zhu,[2] Lei Shao,[3] Tsz Him Chow,[1,] and Jianfang Wang[1,]**

[1]Department of Physics, The Chinese University of Hong Kong, Shatin, Hong Kong SAR 999077, China

[2]State Key Laboratory of Quality Research in Chinese Medicine, Macau Institute for Applied Research in Medicine and Health, Macau University of Science and Technology, Avenida Wai Long, Taipa, Macao SAR 999078, China

[3]State Key Laboratory of Optoelectronic Materials and Technologies, Guangdong Province Key Laboratory of Display Material and Technology, School of Electronics and Information Technology, Sun Yat-sen University, Guangzhou 510275, China

*Email: thchow@phy.cuhk.edu.hk; jfwang@phy.cuhk.edu.hk

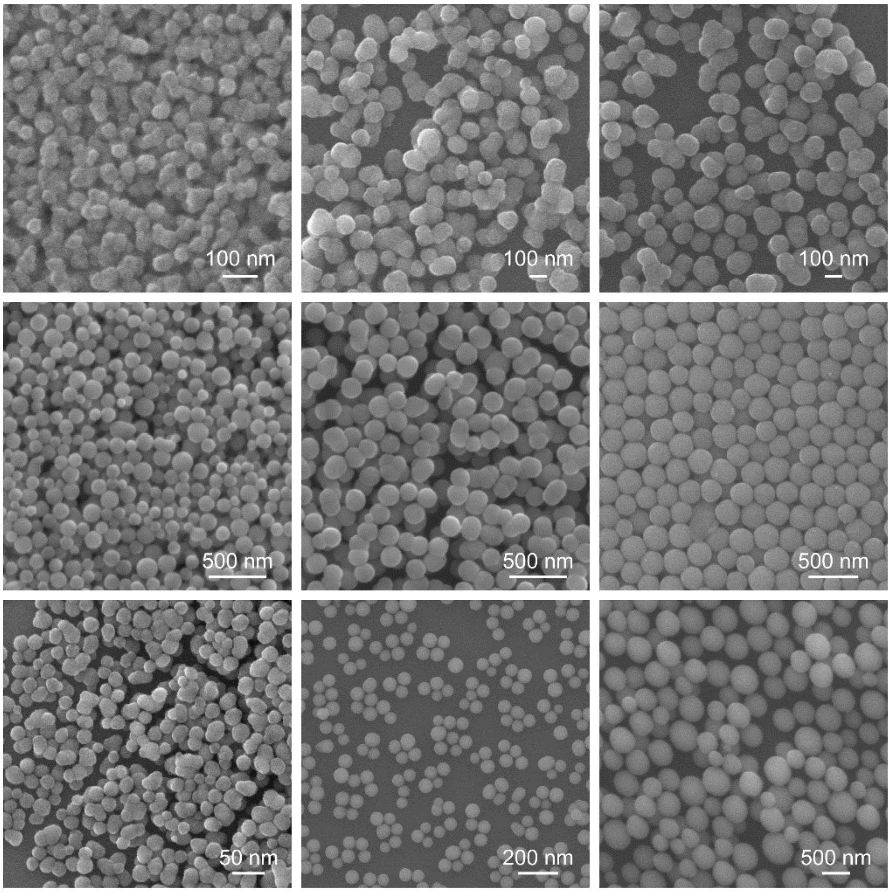

Figure S1. SEM images of the differently sized TiO_2 NSs.

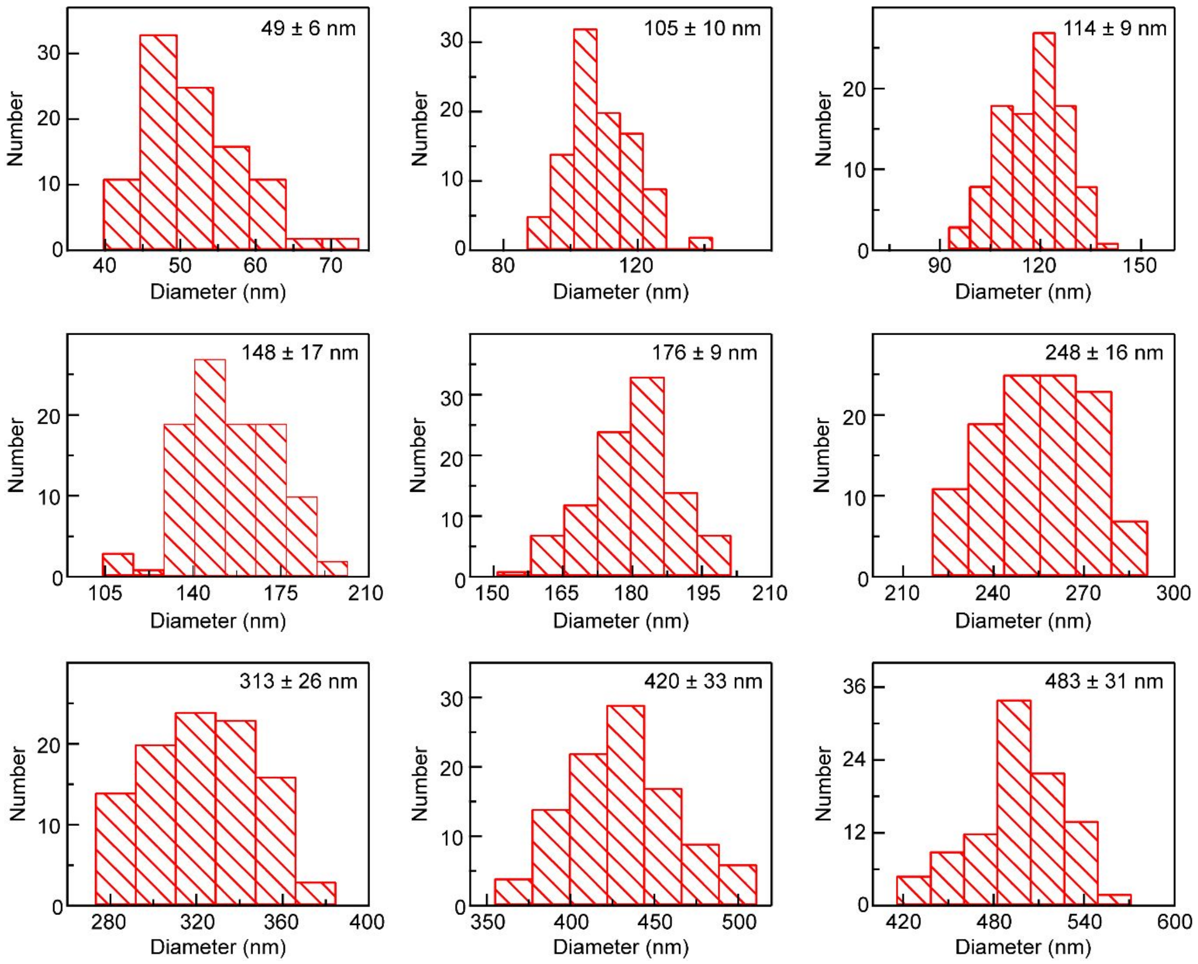

Figure S2. Statistics of the diameters of the TiO_2 NSs. 100 nanoparticles were counted for each sample.

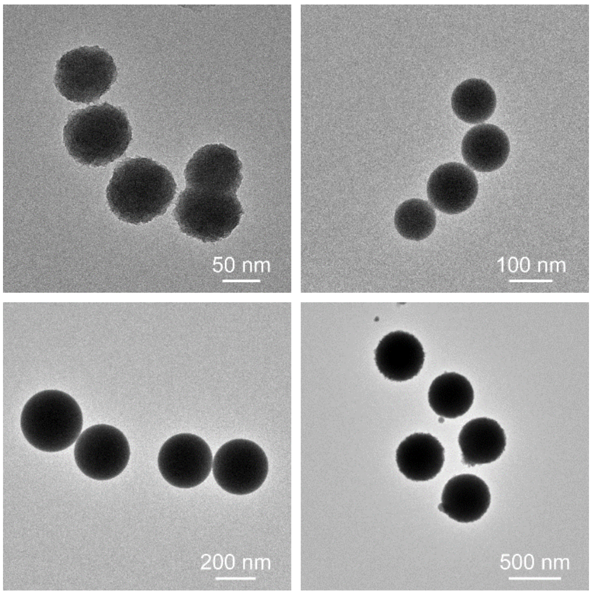

Figure S3. TEM images of the differently sized TiO_2 NSs. The average sizes of the NS samples are 49 ± 6 nm (top left), 114 ± 9 nm (top right), 248 ± 16 nm (bottom left), and 483 ± 31 nm (bottom right).

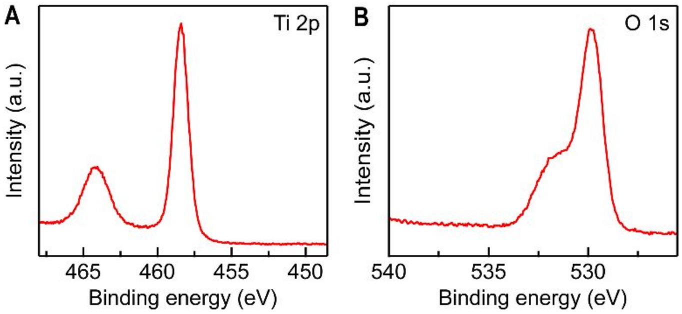

Figure S4. XPS spectra of the TiO_2 NS sample. (A) Ti 2p. (B) O 1s.

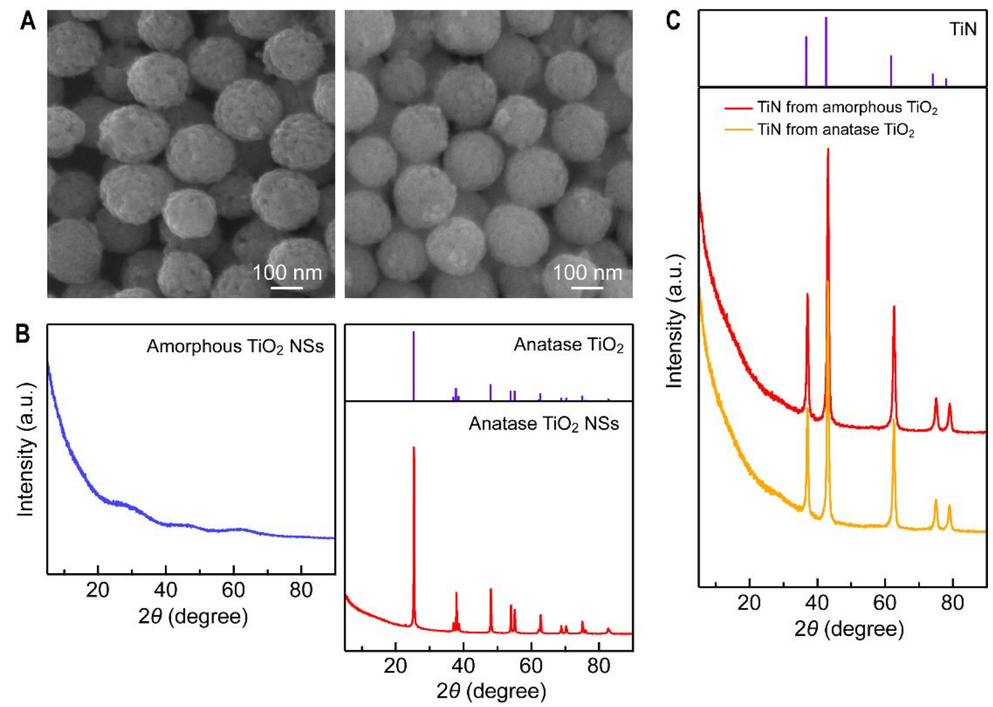

Figure S5. Nitridation of the TiO_2 NSs by two different methods. (A) SEM images of the TiN NSs produced through the nitridation treatment of the amorphous (left) and anatase (right) TiO_2 NSs. (B**)** XRD patterns of the amorphous (left) and anatase (right) TiO_2 NSs before nitridation treatment. The standard XRD pattern (JCPDS No. 84-1285) of anatase TiO_2 is also given for comparison. (C) XRD patterns of the TiN NS samples produced through nitridation of the amorphous and anatase TiO_2 NSs, respectively. The standard XRD pattern (JCPDS No. 87-0628) of TiN is also given for comparison.

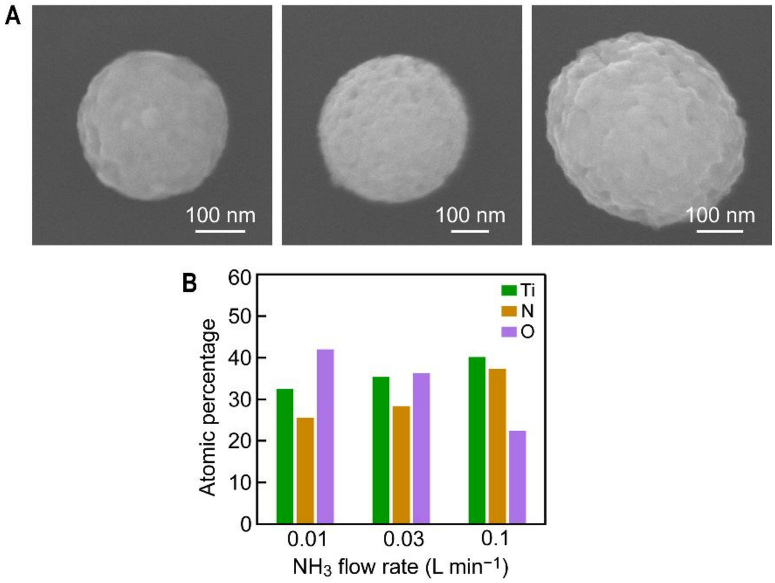

Figure S6. Morphology and composition of the TiN NSs. (A) SEM images of the TiN NSs prepared by nitridation with the three different ammonia flow rates. (B) Elemental compositions obtained from EDX for the three samples.

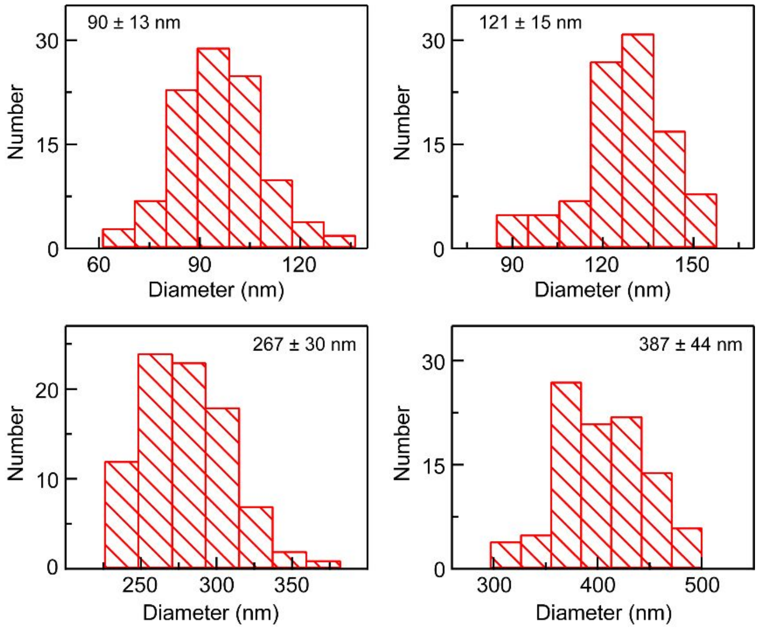

Figure S7. Statistics of the diameters of the TiN NSs. 100 particles were counted for each sample.

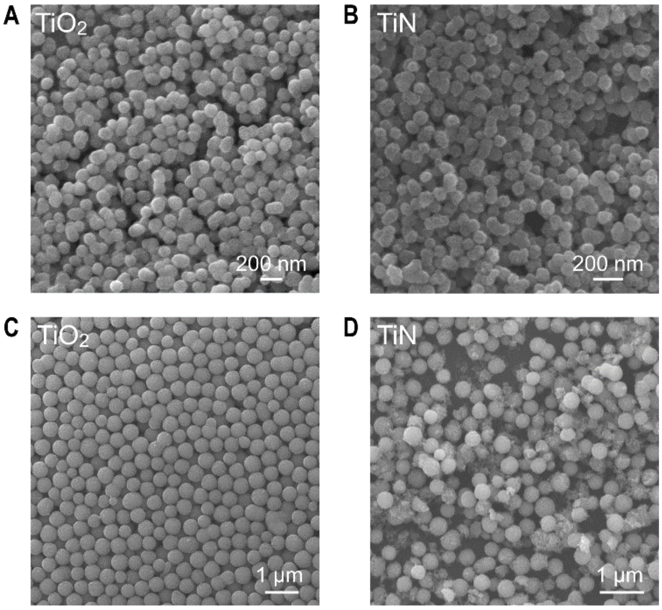

Figure S8. Shrinkage of the TiN NSs induced by nitridation. (A,B) SEM images of the TiO_2 NSs before (A) and after (B) nitridation. The average diameters are 141 ± 10 nm (A) and 87 ± 7 nm (B). (C,D) SEM images of the TiO_2 NSs before (C) and after (D) nitridation. The average diameters are 494 ± 27 nm (C) and 375 ± 32 nm (D).

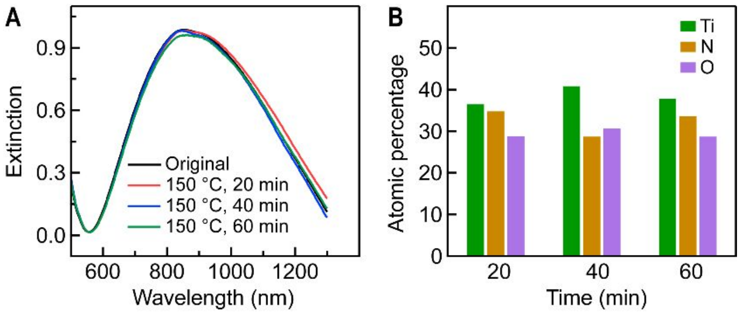

Figure S9. Thermal treatment of the TiN NSs at 150 °C in air. (A) Extinction spectra of the TiN NSs after the thermal treatment for different periods of time. (B) Elemental compositions of the TiN NSs thermally treated for different periods of time. The average diameter of the TiN NS sample is 121 nm.

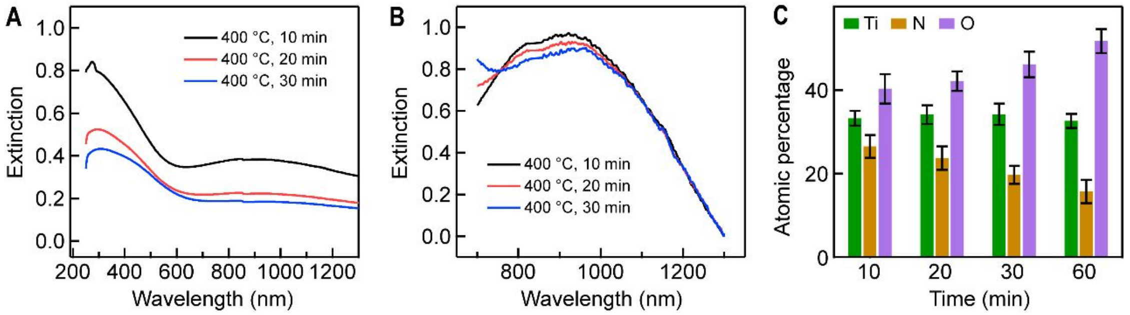

Figure S10. Heat treatment of the TiN NS sample at 400 °C in air. (A) Extinction spectra of the TiN NSs thermally treated for different periods of time. (B) Extinction spectra normalized against the extinction peak thermally treated for 10 min. (C) Elemental compositions of the TiN NSs thermally treated for different periods of time. The average diameter of the TiN NSs is 121 nm.

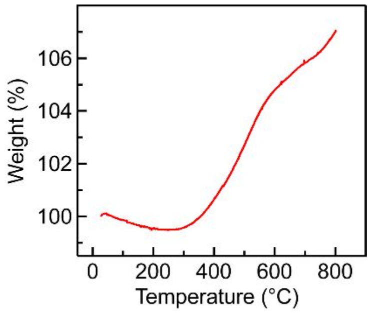

Figure S11. TGA trace of the 121 nm TiN NSs.

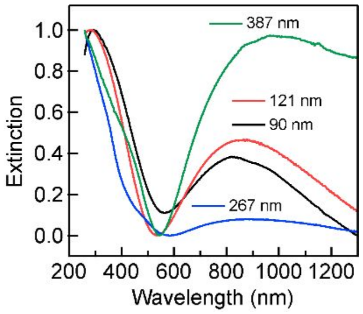

Figure S12. Extinction spectra of the four differently sized TiN NS samples in the spectral range from 260 nm to1300 nm.

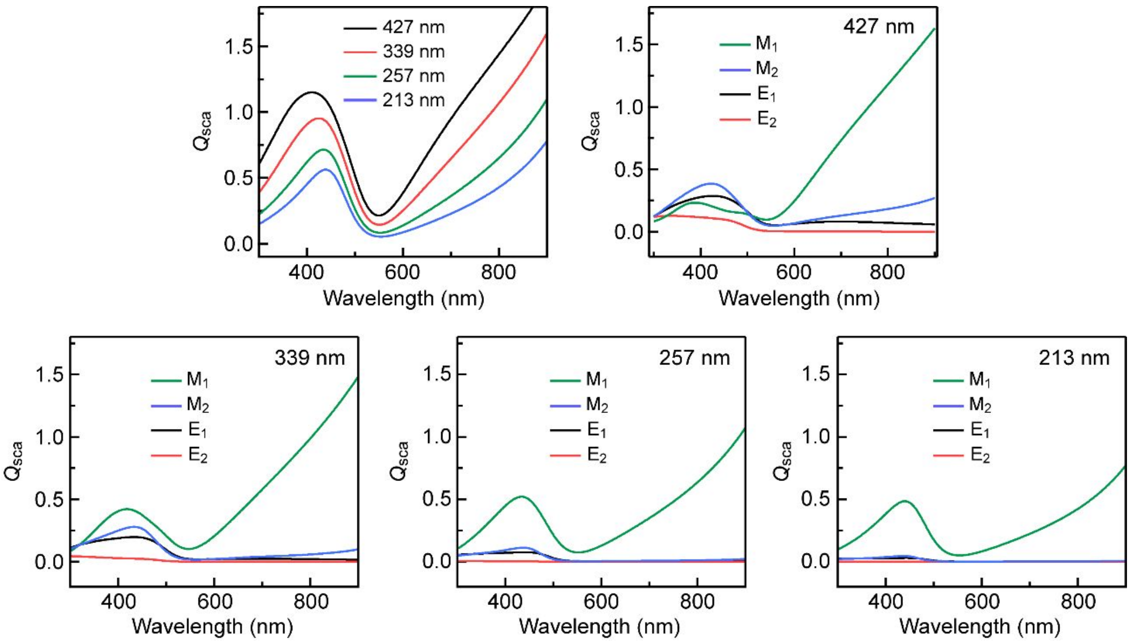

Figure S13. Calculated scattering efficiency spectra of the TiN NSs. The calculations were based on the Drude–Lorentz model. Four diameters were considered. E1 and E2 denote the electric dipole and quadrupole resonance modes, respectively. M1 and M2 denote the magnetic dipole and quadrupole resonance modes, respectively.

The Drude–Lorentz model is expressed as

$$\varepsilon(\omega) = \varepsilon_\infty - \frac{\omega_\mathrm{p}^2}{\omega^2 - i\omega r} + \sum_i \frac{f_i\omega_\mathrm{p}^2}{\omega_{0i}^2 - \omega^2 - i\omega\gamma_i} \quad (1)$$

where the dielectric function $\varepsilon(\omega)$ is a function of the angular frequency ω

$$\omega = 2\pi\nu = 2\pi c/\lambda \quad (2)$$

The parameters involved in the equations above are: the high-frequency permittivity ε_∞; the plasma frequency ω_p in the Drude term; the damping coefficient γ in the Drude term; the oscillator strength of the ith Lorentz oscillator f_i; the resonance frequency of the ith Lorentz oscillator ω_{0i}; and the damping factor of the ith Lorentz oscillator γ_i. The plasma frequency ω_p and resonance frequency ω_{01} are related to the plasma energy (E_p) and resonance energy (E_1) according to

$$E = \hbar\omega \quad (3)$$

where $\hbar$ is the reduced Plank constant. The plasma frequency is determined by

$$\omega_\mathrm{p} = \sqrt{\frac{ne^2}{\varepsilon_0 m_\mathrm{e}^*}} \quad (4)$$

where n is the free electron density, e is the elementary charge, ε_0 is the vacuum permittivity, and m_e^* is the electron effective mass.

Note that the above equation gives the general form of the Drude-Lorentz model, which is applicable for cases with an arbitrary number of Lorentz oscillators. Since there is only one Lorentz oscillator in our work, the expression is simplified as

$$\varepsilon(\omega) = \varepsilon_{\infty} - \frac{\omega_p^2}{\omega^2 - i\omega\gamma} + \frac{f_1\omega_p^2}{\omega_{01}^2 - \omega^2 - i\omega\gamma_1} \quad (5)$$

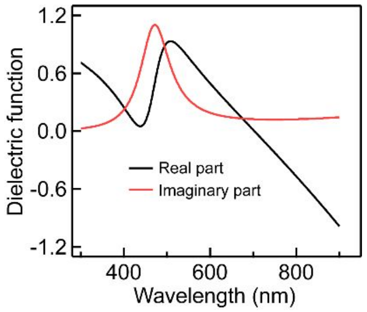

Figure S14. Modelled dielectric function of TiN. The dielectric function was modelled based on the Drude-Lorentz model. In the Drude term, the high-frequency permittivity ε_{∞}, plasma energy E_p, and damping factor γ are 1.06 eV, 2.11 eV, and 0.47 eV, respectively. These values are close to those of reported TiN films.[1] The parameters for the Lorentz term are: the oscillator strength, 0.30; the resonance energy E_1, 2.63 eV; and the damping factor γ_1, 0.47 eV.

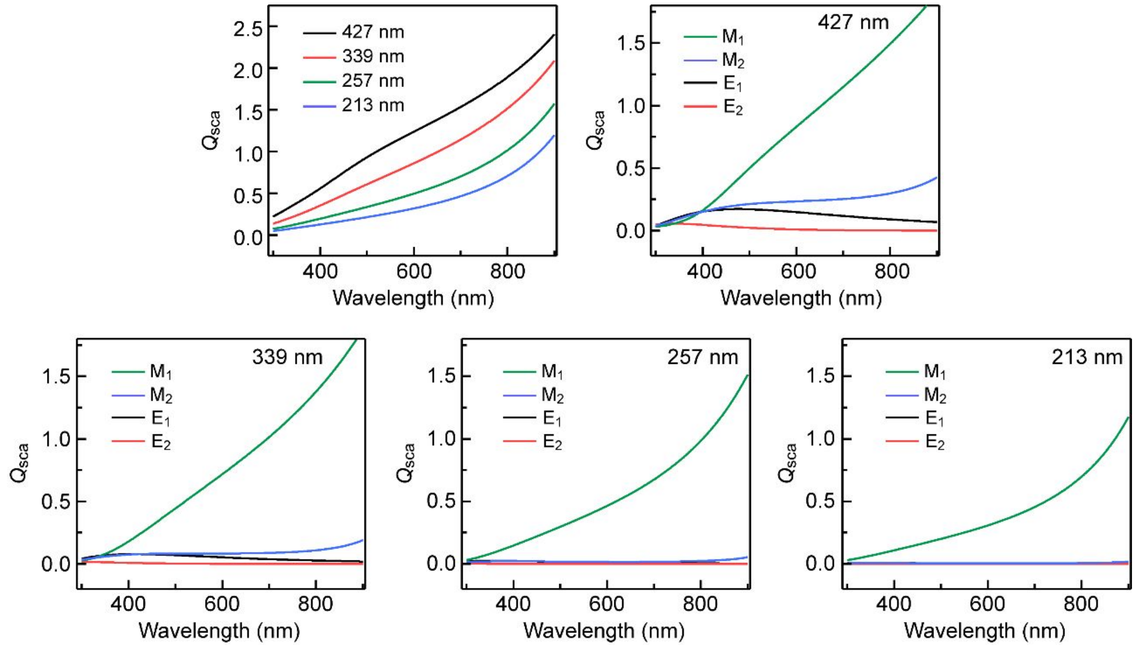

Figure S15. Calculated scattering efficiency spectra of the TiN NSs. The calculations were based on the Drude model only.

In the Drude model, the dielectric function $\varepsilon(\omega)$ is expressed as a function of the angular frequency ω

$$\varepsilon(\omega) = \varepsilon_{\infty} - \frac{\omega_p^2}{\omega^2 - i\omega\gamma} \tag{6}$$

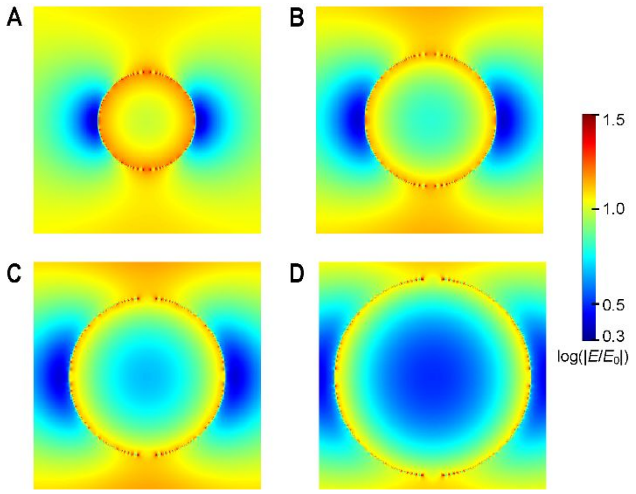

Figure S16. Electric field enhancement of the four differently sized TiN NSs. (A) 213 nm in diameter. (B) 285 nm in diameter. (C) 339 nm in diameter. (D) 427 nm in diameter. The field distributions were simulated at the respective plasmon resonance wavelengths of the TiN NSs. Their plasmon resonance wavelengths are 655 nm, 665 nm, 675 nm, and 697 nm, respectively. The excitation light is polarized along the vertical direction.

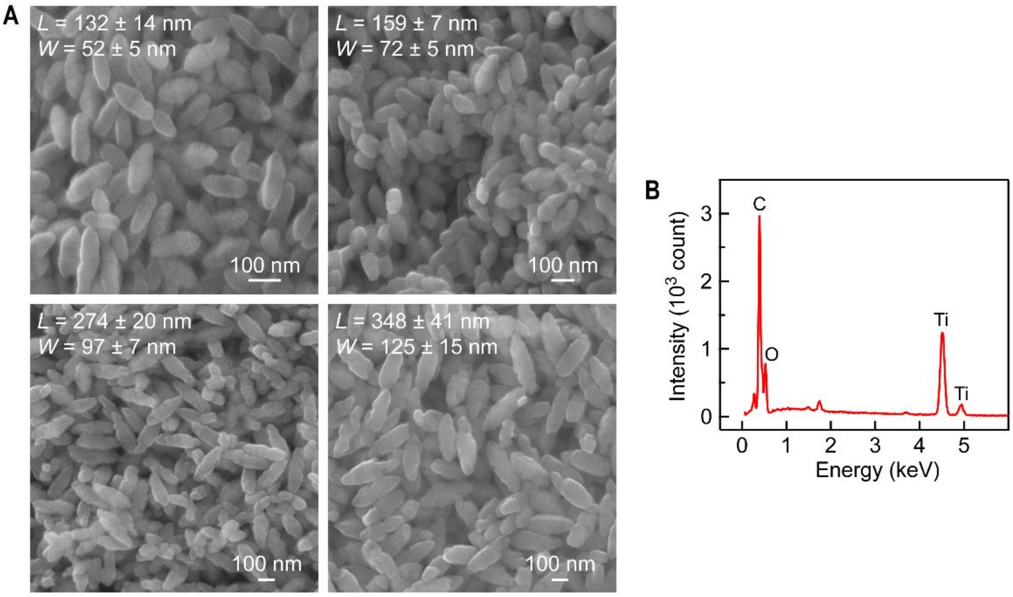

Figure S17. TiO_2 NBP samples. (A) SEM images of the TiO_2 NBP samples with different sizes. (B) Representative EDX spectrum for one TiO_2 NBP sample. The average Ti to O atomic ratio is (35 ± 3) : (64 ± 3). The C and Cu signals in the spectrum are from the TEM grid.

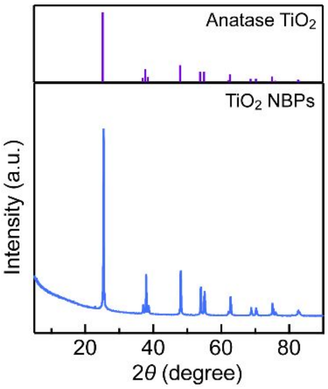

Figure S18. XRD pattern of a representative TiO_2 NBP sample. The peaks can be indexed according to the standard XRD pattern (JCPDS No. 84-1285) of anatase TiO_2.

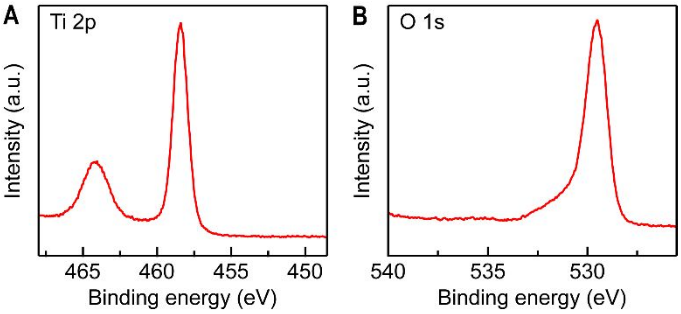

Figure S19. XPS spectra of a representative TiO_2 NBP sample. (A) Ti 2p. (B) O 1s.

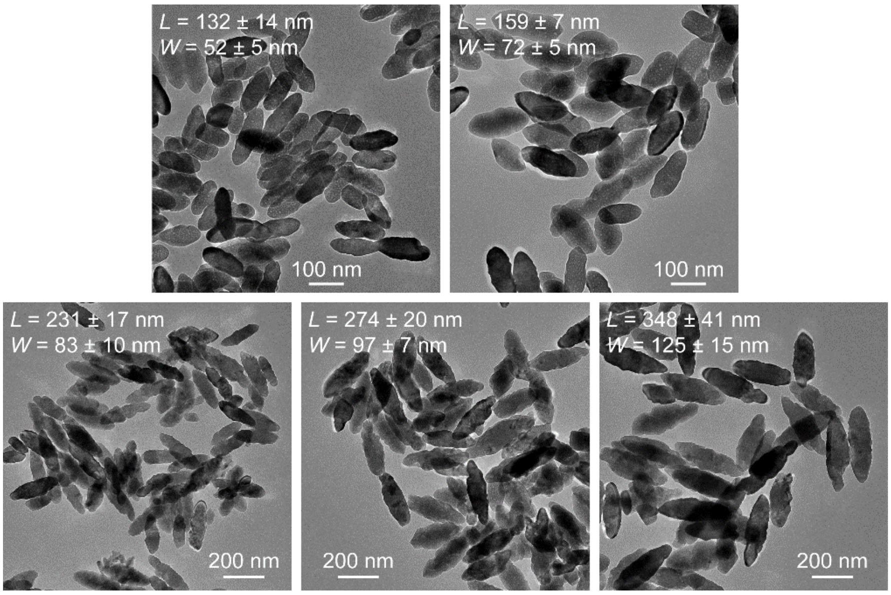

Figure S20. TEM images of the TiO_2 NBP samples with different sizes.

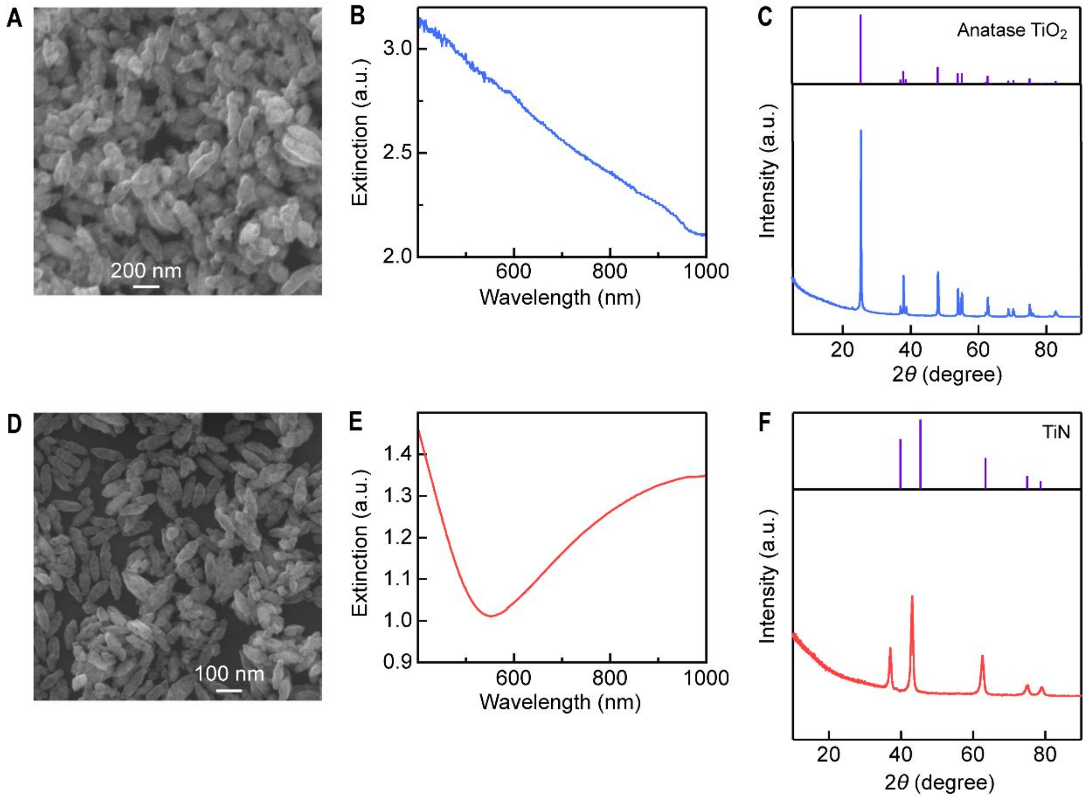

Figure S21. Nitridation of the differently sized TiO_2 NPBs. (A–C) SEM image (A), extinction spectrum (B), and XRD pattern (C) of the nitridized product. The sizes of the TiO_2 NBPs are L = 348 ± 41 nm and W = 125 ± 15nm. The standard XRD pattern (JCPDS No. 84-1285) of anatase TiO_2 is also given for comparison. (D–F) SEM image (D), extinction spectrum (E), and XRD pattern (F) of the nitridized product. The sizes of the TiO_2 NBPs are L = 274 ± 20 nm and W = 97 ± 7 nm. The standard XRD pattern (JCPDS No. 87-0628) of TiN is also given for comparison. The ammonia flow rate was 0.03 L min^{-1}.

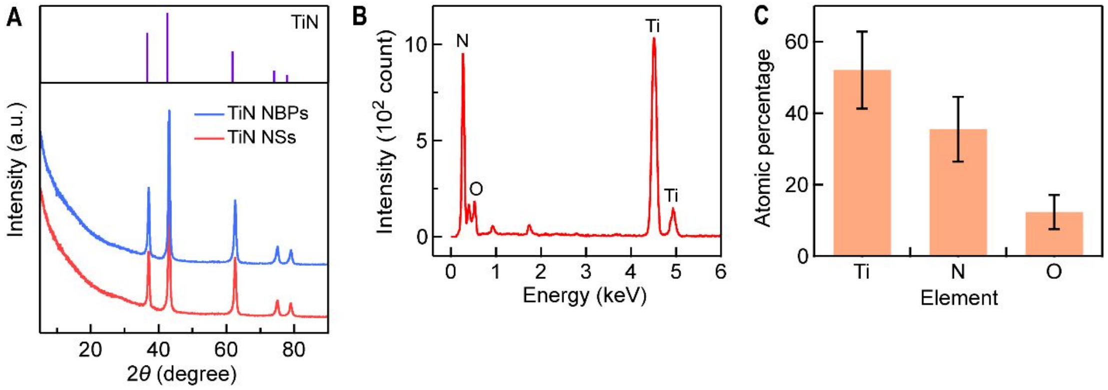

Figure S22. Characterization of the TiN NBPs. (A) XRD patterns of one representative TiN NS sample and one representative TiN NBP sample. The standard XRD pattern (JCPDS No. 87-0628) of TiN is also provided for comparison. (B) EDX spectrum of a representative TiN NBP sample. (C) Elemental composition of a representative TiN NBP sample.

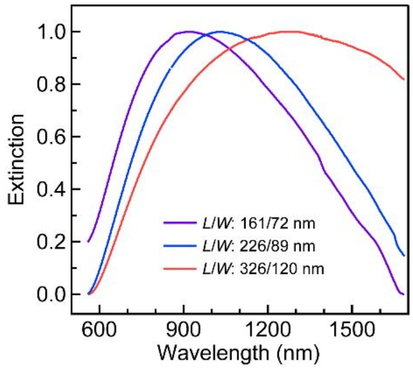

Figure S23. Normalized extinction spectra of the differently sized TiN NBP samples. They were dispersed in D_2O. The transverse plasmon wavelengths of the three samples are 910 nm, 1030 nm, and 1280 nm, respectively.

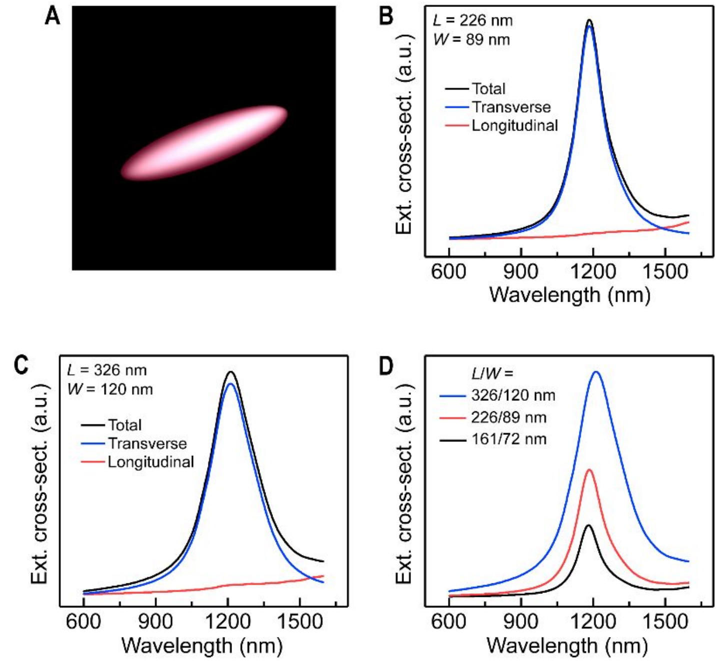

Figure S24. FDTD-simulated extinction spectra of the TiN NBPs. (A) Structure model used in the simulations. (B,C) Simulated extinction cross-section spectra of the two differently sized TiN NBPs. (D) Comparison of the simulated extinction cross-section spectra of the three differently sized TiN NBPs. The transverse plasmon wavelengths of the three TiN NBPs are 1180 nm, 1185 nm, and 1210 nm, respectively. The longitudinal excitation direction is parallel to the long axis of the TiN NBP, and the transverse excitation direction is perpendicular to the long axis of the TiN NBP.

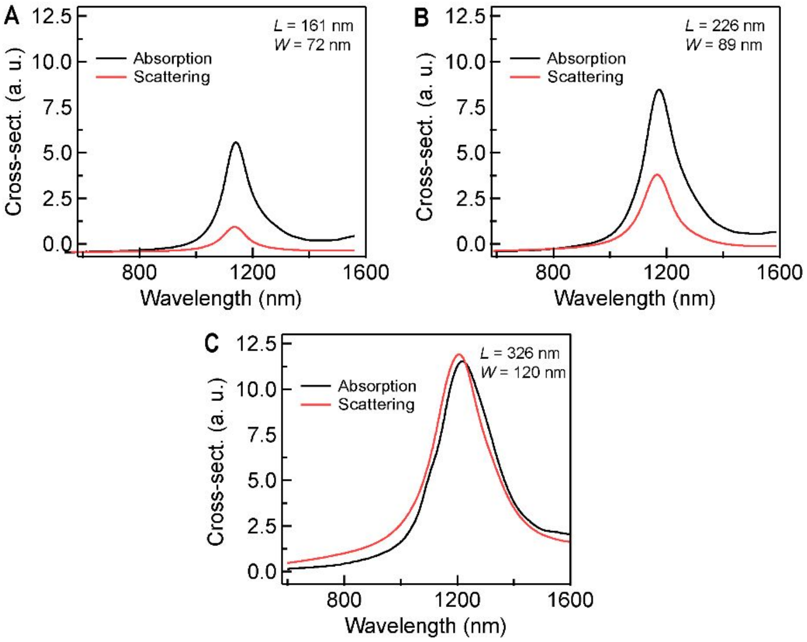

Figure S25. FDTD-simulated absorption and scattering spectra for the three differently sized TiN NBPs. The excitation light is polarized along the length axis of the NBP.

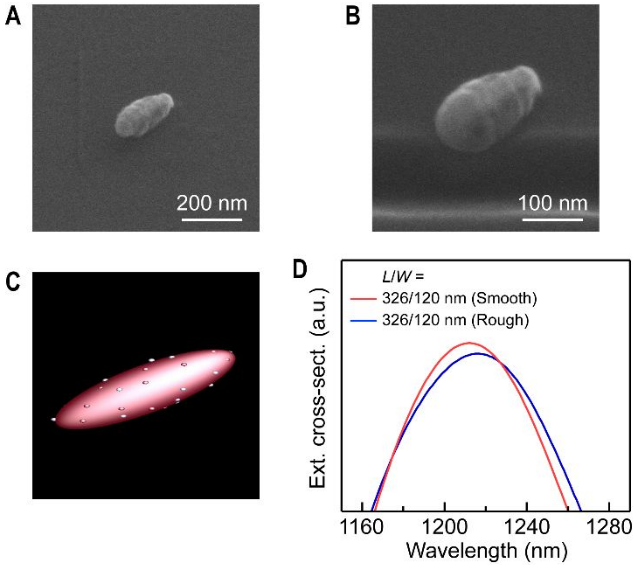

Figure S26. Examination of the effect of the surface roughness. (A) SEM image of a representative TiN NBP. (B) SEM image of the TiN NBP after being cut at the waist. (C) Model structure used for the FDTD simulation (white spheres: TiN, 10 nm in diameter; light purple spherical cavities: removed portions of TiN and filled with air, 10 nm in diameter). (D) Simulated extinction cross-section spectra of the rough TiN NBP in (C) and the smooth TiN NBP with the same size.

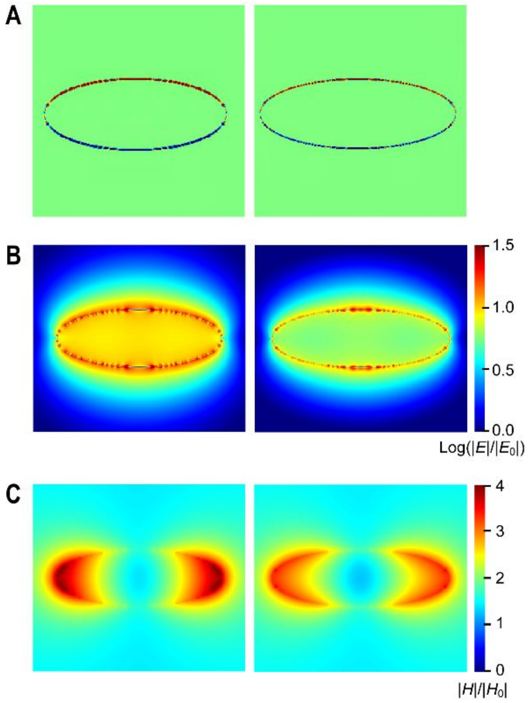

Figure S27. Simulated charge distribution and electromagnetic field enhancement contours. (A) Charge distribution contours. (B) Electric field enhancement contours. (C) Magnetic field enhancement contours. The TiN NBPs are under transverse excitation. The sizes of the left and right TiN NBPs are L = 226 nm, W = 89 nm and L = 326 nm, W = 120 nm, respectively.

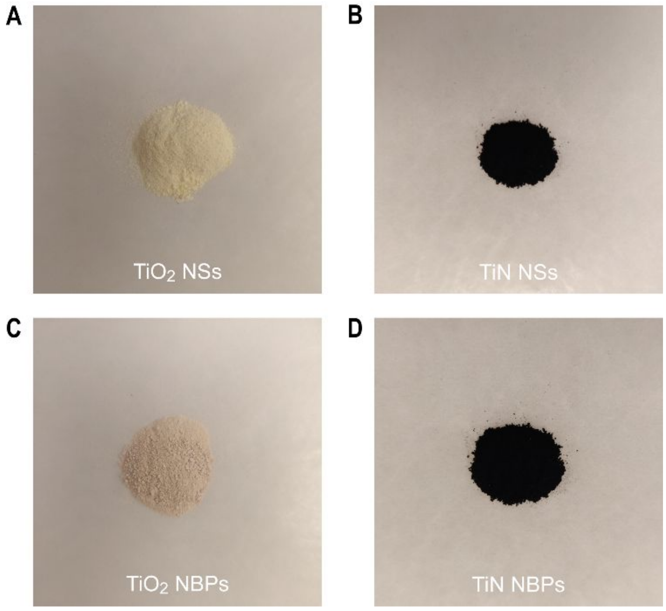

Figure S28. Gram-scale synthesis of the TiN nanoparticles. (A,B) Photographs of the TiO_2 NSs (A, diameter: 141 ± 10 nm) and the corresponding TiN NSs (B, diameter: 87 ± 7 nm). (C,D) Photographs of the TiO_2 NBPs (C, length: 375 ± 37 nm, waist width: 135 ± 18 nm) and the corresponding TiN NBPs (D, length: 352 ± 33 nm, waist width: 131 ± 13 nm).

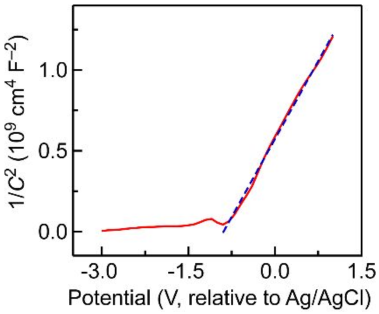

Figure S29. Mott–Schottky plot of a representative TiN NBP sample. The measurement was made at the frequency of 1000 Hz.

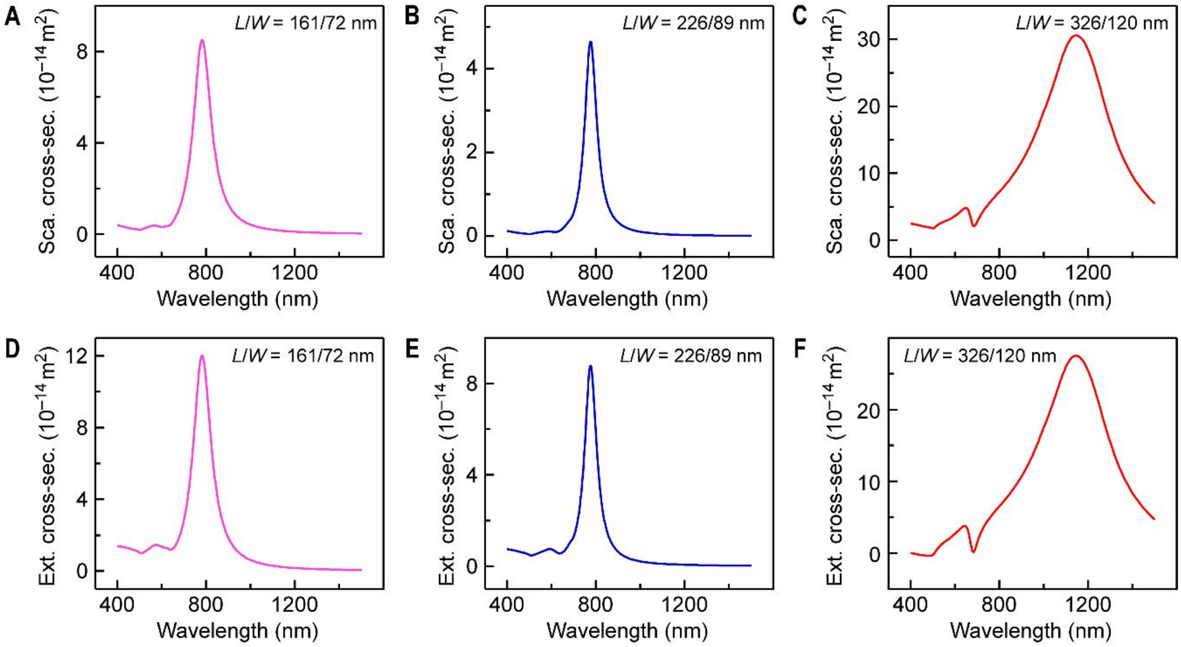

Figure S30. Simulation of the Au NBPs. (A–C) Simulated scattering cross-section spectra of the three differently sized Au NBPs. (D–F) Simulated extinction cross-section spectra of the three Au NBPs. The Au NBPs are under longitudinal excitation.

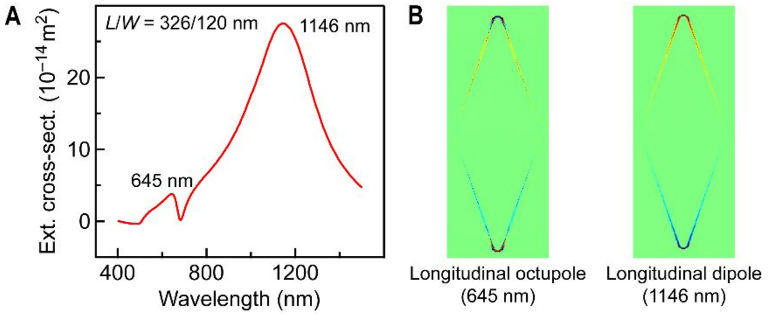

Figure S31. Simulation of the Au NBP. (A) Simulated extinction cross-section spectrum of a representative Au NBP. (B) Charge distribution contours calculated for the plasmon peaks located at 645 nm and 1146 nm, respectively, as indicated in (A).

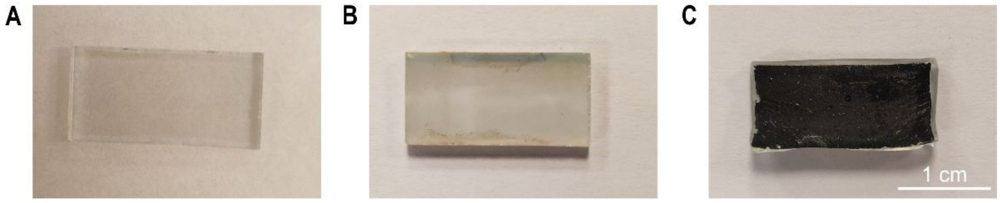

Figure S32. Photographs of the NRAs. (A) Blank FTO substrate. (B) TiO_2 NRA. (C) TiN NRA.

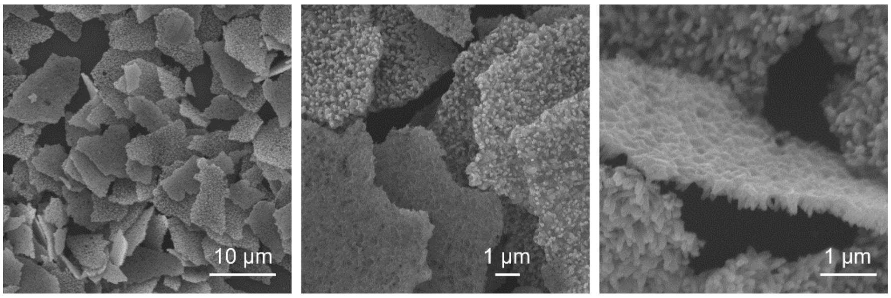

Figure S33. SEM images of the TiN NRA after ultrasonication.

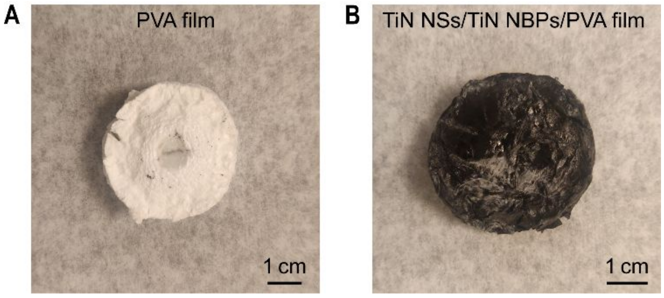

Figure S34. Photographs of the films. (A) PVA film. (B) TiN NSs/TiN NBPs/PVA composite film.

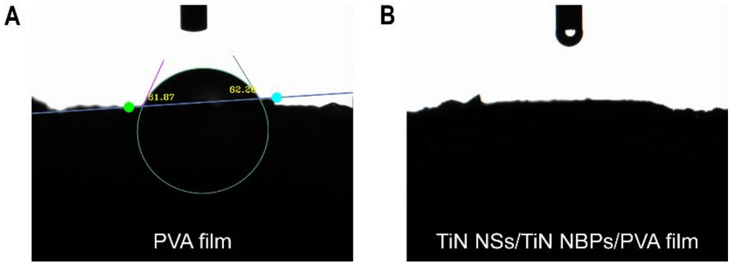

Figure S35. Water contact angle tests. (A,B) Optical images of the water droplets on the pure PVA film (A) and the TiN NSs/TiN NBPs/PVA composite film (B).

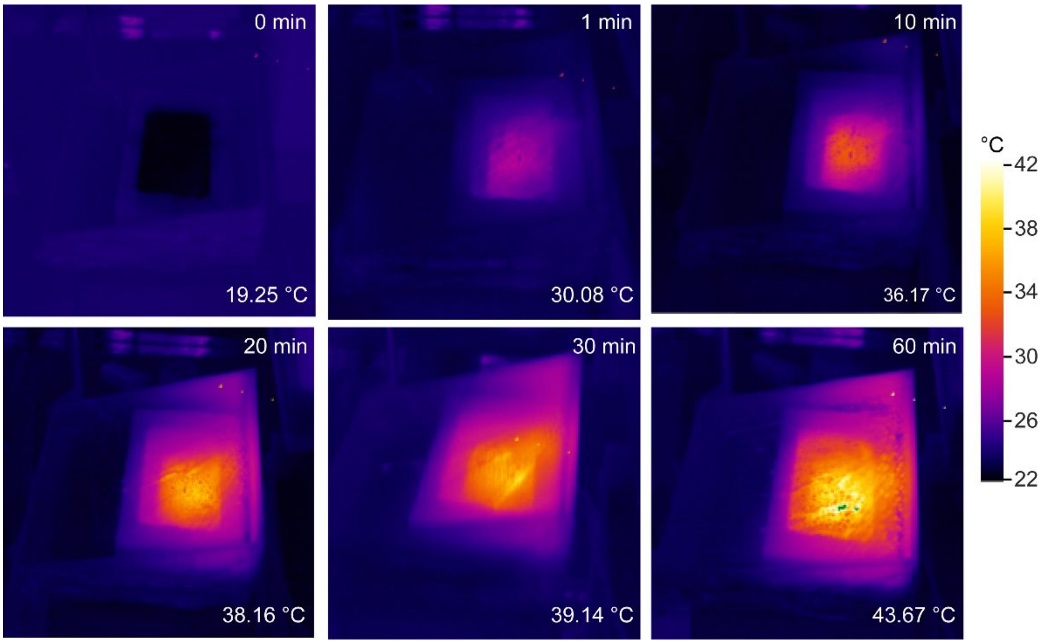

Figure S36. Infrared images. The images were recorded after the simulated sunlight was turned on. They show the temperature rise on the TiN NSs/TiN NBPs/PVA composite film.

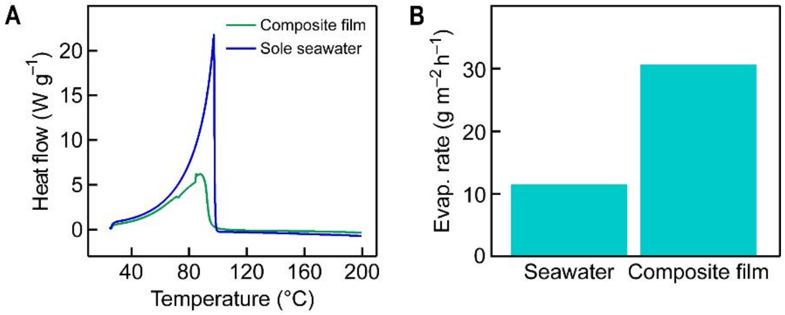

Figure S37. Evaporation enthalpy of seawater. (A) DSC traces of sole seawater and seawater in the presence of the composite film. (B) Evaporation rates of bulk seawater and seawater in the presence of the composite film in the dark.

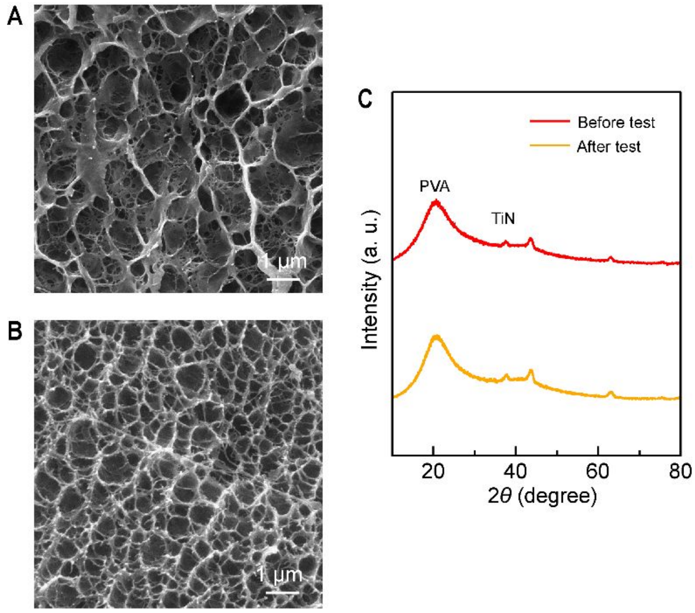

Figure S38. SEM images and XRD patterns of the TiN/PVA composite film. (A,B) SEM images of the TiN/PVA composite film before (A) and after (B) the stability test. (C) XRD patterns of the TiN composite film before and after the stability test.

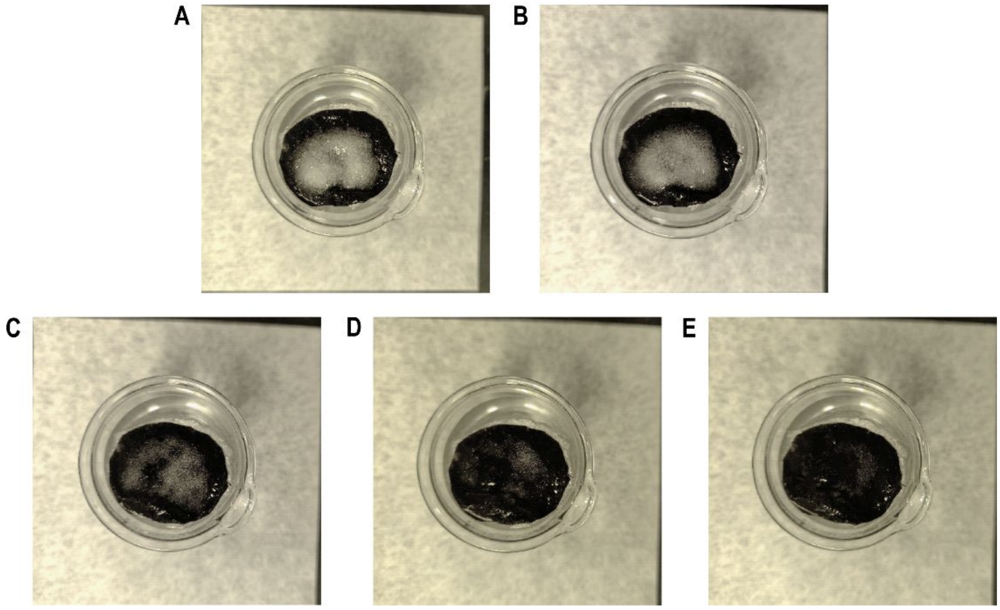

Figure S39. Antifouling performance. (A) The amount of salt produced by the film during the evaporation of seawater in one day was placed on the composite film. (B–E) The sample was then left indoors for the diffusion of the salt on the surface of the film to be monitored over time. The photographs were taken at 0 min (A), 10 min (B), 1 h (C), 3 h (D), and 5 h (E).

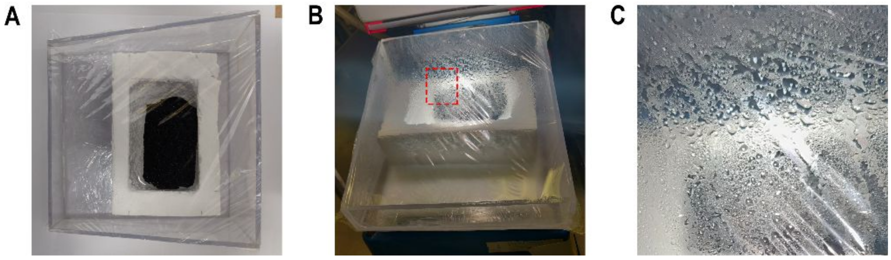

Figure S40. Seawater desalination device. (A) Photograph of the seawater desalination device. (B) Condensation of freshwater droplets on the inner surface of the covering plastic film. (C) Enlarged photograph of the boxed area in (D) showing the formation of water droplets.

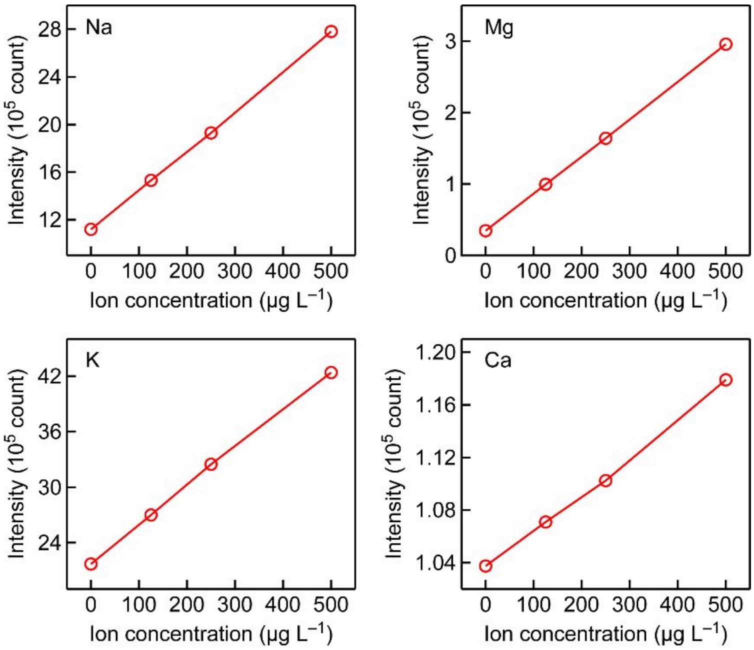

Figure S41. Calibration curves for the ICP-MS measurements. The signal intensities are plotted as functions of the ion concentrations for the different cations.

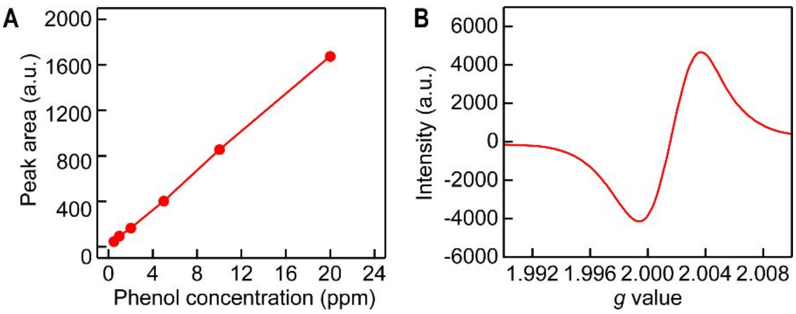

Figure S42. Phenol determination and EPR measurement. (A) Calibration curve for the phenol concentrations in aqueous solutions obtained using liquid chromatography. (B) EPR spectrum of the mixture of the TiN NSs and NBPs.

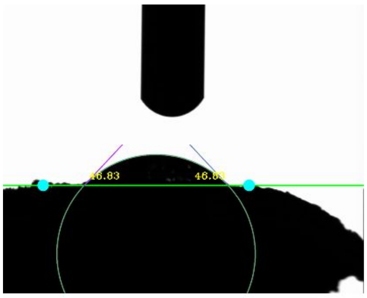

Figure S43. Optical image of a phenol solution droplet on the composite film.

Table S1. Solar Vapor Generation Rates and Solar Energy Conversion Efficiencies of Representative Works on Solar Seawater Desalination

Material	Vapor generation rate (kg m^{-2} h^{-1})	Energy conversion efficiency	Sunlight power (kW m^{-2})	Reference
TiN/AAO	1.1	78	1	2
ZrN/AAO	1.27	88	1	2
HfN/AAO	1.36	95	1	2
MoO_3 nanospheres	2.09	94	1	3
ANCC	1.36	92	1	4
Au nano-trepang/PAN	2.7	79	1	5
Plamonic wood	11.8	85	10	6
Au/D-NPT	5.7	90	4	7
This work	3.8	95	1	

Table S2. Concentrations of the Ions in the Desalinated Water

Element	Concentration (mg L^{-1})	Element	Concentration (mg L^{-1})	Element	Concentration (mg L^{-1})
Sc	0	Tb	0.0006	Fe	0.0057
Be	0	Pd	0.0007	Te	0.0059
Tm	0	Bi	0.0008	Nb	0.0066
Yb	0.0001	Pt	0.0008	Sn	0.0075
Zr	0.0001	Gd	0.0009	Pb	0.0088
Ag	0.0002	Ta	0.0009	In	0.009
Y	0.0002	Eu	0.0011	V	0.0115
Lu	0.0002	Er	0.0015	Ru	0.0115
Ti	0.0002	Ce	0.0033	Mn	0.0123
La	0.0002	Cu	0.0039	Sb	0.0142
Cr	0.0004	Ir	0.0044	Hf	0.0145
Rh	0.0005	Sm	0.0051	Li	0.0147
Cd	0.0005	W	0.0006	Ga	0.0159
Ho	0.0006	Co	0.0007	Nd	0.0162
Au	0.0006	TI	0.0008	Hg	0.0166
Dy	0.0006	Pr	0.0008	Se	0.0178
Ni	0.0179	Al	0.0189	P	0.0213
Ge	0.0249	Mo	0.0532	Sr	0.0693
As	0.0973	Ba	0.1273	Zn	0.133
Na	25.01	Si	0.2759	B	0.4577
K	2.8551	Mg	5.2328	S	5.3433
Ca	6.4725				

Movie S1. Flexibility and tenacity test of the TiN NSs/TiN NBPs/PVA composite film.

Movie S2. Buoyancy test of the TiN NSs/TiN NBPs/PVA composite film.

Movie S3. Contact angle test of the PVA film.

Movie S4. Contact angle test of the TiN NSs/TiN NBPs/PVA composite film.

SUPPORTING REFERENCES

(1) Naik, G. V.; Shalaev, V. M.; Boltasseva, A. Alternative Plasmonic Materials: beyond Gold and Silver. *Adv. Mater.* **2013**, *25*, 3264–3294.

(2) Traver, E.; Karaballi, R. A.; Monfared, E. Y.; Daurie, H.; Gagnon, A. G.; Dasog, M. TiN, ZrN, and HfN Nanoparticles on Nanoporous Aluminum Oxide Membranes for Solar-driven Water Evaporation and Desalination. *ACS Appl. Nano Mater.* **2020**, *3*, 2787–2794.

(3) Bai, H. Y.; Hu, J. T.; Lam, S. H.; Guo, Y. Z.; Zhu, X. M.; Yang, Z.; Wang, J. F. Turning Dielectric MoO_3 Nanospheres from White to Black through Doping for Efficient Solar Seawater Desalination. *ACS Mater. Lett.* **2022**, *4*, 1584–1592.

(4) Qiao, P. Z.; Wu, J. X.; Li, H. Z.; Xu, Y. C.; Ren, L. P.; Lin, K.; Zhou, W. Plasmon Ag-promoted Solar–Thermal Conversion on Floating Carbon Cloth for Seawater Desalination and Sewage Disposal. *ACS Appl. Mater. Interfaces* **2019**, *11*, 7066–7073.

(5) Huang, Z. M.; Li, S. L.; Cui, X.; Wan, Y. P.; Xiao, Y. F.; Tian, S.; Wang, H.; Li, X. Z.; Zhao, Q.; Lee, C-S. A Broadband Aggregation-independent Plasmonic Absorber for Highly Efficient Solar Steam Generation. *J. Mater. Chem. A* **2020**, *8*, 10742–10746.

(6) Zhu, M. W.; Li, Y. J.; Chen, F. J.; Zhu, X. Y.; Dai, J. Q.; Li, Y. F.; Yang, Z.; Yan, X. J.; Song, J. W.; Wang, Y. B.; Hitz, E.; Luo, W.; Lu, M. H.; Yang, B.; Hu, L. B. Plasmonic Wood for High-efficiency Solar Steam Generation. *Adv. Energy Mater.* **2018**, *8*, 1701028.

(7) Zhou, L.; Tan, Y. L.; Ji, D. X.; Zhu, B.; Zhang, P.; Xu, J.; Gan, Q. Q.; Yu, Z. F.; Zhu, J. Self-assembly of Highly Efficient, Broadband Plasmonic Absorbers for Solar Steam Generation. *Sci. Adv.* **2016**, *2*, e1501227.
